# Cross-Country Individual Participant Analysis of 4.1 Million Singleton Births in 5 Countries with Very High Human Development Index Confirms Known Associations but Provides No Biologic Explanation for 2/3 of All Preterm Births

**DOI:** 10.1371/journal.pone.0162506

**Published:** 2016-09-13

**Authors:** David M. Ferrero, Jim Larson, Bo Jacobsson, Gian Carlo Di Renzo, Jane E. Norman, James N. Martin, Mary D’Alton, Ernesto Castelazo, Chris P. Howson, Verena Sengpiel, Matteo Bottai, Jonathan A. Mayo, Gary M. Shaw, Ivan Verdenik, Nataša Tul, Petr Velebil, Sarah Cairns-Smith, Hamid Rushwan, Sabaratnam Arulkumaran, Jennifer L. Howse, Joe Leigh Simpson

**Affiliations:** 1 Boston Consulting Group, Boston, MA, United States of America; 2 March of Dimes Foundation, White Plains, NY, United States of America; 3 International Federation of Gynecology and Obstetrics, London, United Kingdom; 4 Department of Obstetrics and Gynecology, Institute for the Health of Women and Children, Sahlgrenska Academy, University of Gothenburg, Sahlgrenska University Hospital, Gothenburg, Sweden; 5 Department of Pediatrics, Stanford University School of Medicine, Palo Alto, CA, United States of America; 6 Division of Maternal-Fetal Medicine, University of Mississippi, Medical Center, Jackson, MS, United States of America; 7 Department of Obstetrics and Gynecology, Columbia University / College of Physicians and Surgeons, New York, NY, United States of America; 8 Tommy’s Centre for Fetal and Maternal Health, Medical Research Council Centre for Reproductive Health, Queen’s Medical Research Institute, The University of Edinburgh, Edinburgh, United Kingdom; 9 Unit of Biostatistics, IMM, Karolinska Institutet, Stockholm, Sweden; 10 Department of Obstetrics and Gynecology, University of Perugia, Perugia, Italy; 11 Department of Perinatology, Division of Obstetrics and Gynecology, University Medical Centre Ljubljana, Ljubljana, Slovenia; 12 Institute for the Care of Mother and Child, Perinatal Centre, Prague, Czech Republic; 13 Department of Genes and Environment, Division of Epidemiology, Norwegian Institute of Public Health, Oslo, Norway; Universite de Montreal, CANADA

## Abstract

**Background:**

Preterm birth is the most common single cause of perinatal and infant mortality, affecting 15 million infants worldwide each year with global rates increasing. Understanding of risk factors remains poor, and preventive interventions have only limited benefit. Large differences exist in preterm birth rates across high income countries. We hypothesized that understanding the basis for these wide variations could lead to interventions that reduce preterm birth incidence in countries with high rates. We thus sought to assess the contributions of known risk factors for both spontaneous and provider-initiated preterm birth in selected high income countries, estimating also the potential impact of successful interventions due to advances in research, policy and public health, or clinical practice.

**Methods:**

We analyzed individual patient-level data on 4.1 million singleton pregnancies from four countries with very high human development index (Czech Republic, New Zealand, Slovenia, Sweden) and one comparator U.S. state (California) to determine the specific contribution (adjusting for confounding effects) of 21 factors. Both individual and population-attributable preterm birth risks were determined, as were contributors to cross-country differences. We also assessed the ability to predict preterm birth given various sets of known risk factors.

**Findings:**

Previous preterm birth and preeclampsia were the strongest individual risk factors of preterm birth in all datasets, with odds ratios of 4.6–6.0 and 2.8–5.7, respectively, for individual women having those characteristics. In contrast, on a population basis, nulliparity and male sex were the two risk factors with the highest impact on preterm birth rates, accounting for 25–50% and 11–16% of excess population attributable risk, respectively (p<0.001). The importance of nulliparity and male sex on population attributable risk was driven by high prevalence despite low odds ratios for individual women. More than 65% of the total aggregated risk of preterm birth within each country lacks a plausible biologic explanation, and 63% of difference between countries cannot be explained with known factors; thus, research is necessary to elucidate the underlying mechanisms of preterm birth and, hence, therapeutic intervention. Surprisingly, variation in prevalence of known risk factors accounted for less than 35% of the difference in preterm birth rates between countries. Known risk factors had an area under the curve of less than 0.7 in ROC analysis of preterm birth prediction within countries. These data suggest that other influences, as yet unidentified, are involved in preterm birth. Further research into biological mechanisms is warranted.

**Conclusions:**

We have quantified the causes of variation in preterm birth rates among countries with **very high human development index**. The paucity of explicit and currently identified factors amenable to intervention illustrates the limited impact of changes possible through current clinical practice and policy interventions. Our research highlights the urgent need for research into underlying biological causes of preterm birth, which alone are likely to lead to innovative and efficacious interventions.

## Introduction

Preterm birth (birth occurring before 37 weeks of gestation) is an enormous global public health challenge, with 15 million infants born preterm every year and an estimated 35% of deaths in the first 4 weeks of life directly attributable to prematurity [1, 2]. In 2013, preterm birth complications were the leading cause of death of the estimated 6.3 million liveborn children worldwide who died before age 5 years (15.4%), followed by pneumonia (14.9%) and intrapartum-related complications (10.5%) [3]. Preterm birth also exacts a substantial toll on morbidity and on the health economy–as preterm birth costs the U.S. health system at least $26B yearly [4].

The World Health Organization (WHO) "Born Too Soon" report (1) concluded that a 50% reduction of preterm birth specific mortality in resource-poor countries is achievable by 2025. On the other hand, the prevention opportunity is weak based on current proven interventions. Assuming even optimum coverage and historical-level reductions of best-in-class countries, a miniscule 5% rate reduction in preterm birth is possible in 39 countries with very high human development index (VHHDI). This is a shockingly small absolute reduction opportunity (0.5%; from 9.6% to 9.1%) given the substantial disease burden of preterm birth [5].

Risk factors known to be associated with preterm birth include multiple medical, genetic, environmental and socioeconomic factors, not always considered in combination [6–8]. Previously observed risk factors include certain maternal or fetal conditions (e.g. preeclampsia, malformations), previous preterm delivery, multifetal gestation, young or advanced maternal age, assisted reproductive technology (ART) (especially with multifetal gestation), infection, cervical anomalies, certain ethnicities, smoking, extremes of body-mass index (BMI), low socio-economic status and especially early (and often unnecessary) elective delivery due to errors in gestational age dating and other reasons. Other less well-validated risk factors include stress, excessive physical work, sexual activity, alcohol consumption and periodontal disease. Despite much accumulated knowledge on individual etiological factors, the interactions among risk factors and the pathophysiology of preterm birth remain unclear [6].

Important differences in preterm birth rates exist across countries. Among countries with VHHDI, preterm birth rates vary widely, in 2010 from 5.3 per 100 live births in Latvia to 14.7 per 100 live births in Cyprus [5]. Causes behind these wide variations in preterm birth rates are largely unknown. Understanding the causes of this variation has the potential to guide identification and use of impactful interventions.

The purpose of our study was to perform the most robust cross-country analysis of preterm birth etiology undertaken to date, utilizing individual patient-level pregnancy and birth data from select countries with VHHDI to 1) assess the relative contributions of known risk factors of preterm birth to individual preterm birth risk, 2) assess the impact of each factor on population preterm birth rates (taking into account relative risk and prevalence of the risk factor), 3) estimate the potential impact of three key intervention areas (i.e., research, policy and public health, clinical practice) and 4) explain variability of preterm birth rates across selected countries with VHHDI. A secondary purpose was to perform a systematic review of multivariate studies of preterm birth risk factors in order to situate our analysis.

## Methods

**Fig 1:**
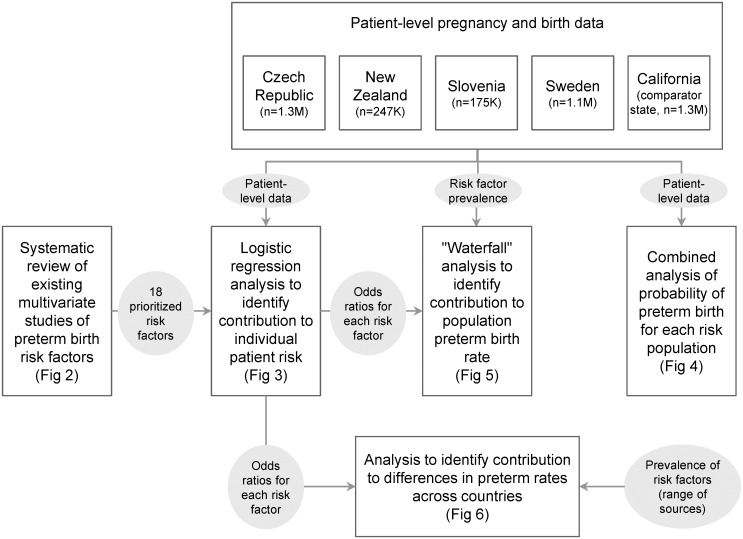
Overview of analytic approach. The four country datasets (Czech Republic, New Zealand, Slovenia, Sweden) and the U.S. comparator state (California) are indicated at the top of the figure along with the number of births included in the analysis from each. Each of the five core analyses are represented in the boxes below, and the relationship between analyses is represented by arrows.

We conducted five distinct and interrelated analyses as part of this effort. See Fig 1 for an overview of our analytic approach.

### Literature search of previously published multivariate analyses of preterm birth

We performed a literature search of multivariate studies of preterm birth risk factors published since 1985 using the combination of key words "premature", "preterm" and "risk", in Web of Science (Thomson Reuters) and MEDLINE (United States National Library of Medicine). This search yielded ~11,500 search results, following which we excluded studies that considered fewer than 6 non-clinical maternal or fetal risk factors as covariates (e.g., maternal age, education, poverty). We included all risk factors considered by at least 3 studies. Where categorical variables were reported, we reported the category having the largest significant odds ratio (e.g. BMI > 30 for the category of maternal weight).

### Individual patient risk vs. population preterm birth rate

We use two terms throughout this study to describe the contribution of preterm birth risk factors. 1) *Individual patient risk* refers to the increased likelihood of giving birth preterm for an individual woman with the risk factor. 2) *Population attributable risk* refers to the contribution of that risk factor to the overall population preterm birth rate, and is a function of both the increase in risk when the risk factor is present and the prevalence of the risk factor in that population. *Individual patient risk* is relevant to an individual patient as well as to obstetricians and midwives seeking to understand and estimate a given patient's risk and determine appropriate clinical care given the presence of a set of risk factors. *Population preterm birth rate* by contrast is relevant to policymakers and public health clinicians seeking to reduce the overall preterm birth rate.

### Individual patient-level data sources

Based on published literature, internet searches and prior knowledge of the authors, we identified a convenient sample of international individual patient-level data sources with at least 5 years of pregnancy outcome data, encompassing at least 10 clinical and non-clinical variables. Based on data robustness (i.e., datasets previously described) and access provisions (i.e., being able to access the data within 4 months of request), we included individual patient-level datasets from four countries (Czech Republic, New Zealand, Slovenia, Sweden).

Data sources were as follows: Czech Republic, the National Registry of Reproduction Health—Mothers at Childbirth and Newborns (http://www.uzis.cz/en); New Zealand, the National Maternity Collection maintained by the New Zealand Ministry of Health (www.health.govt.nz/); Slovenia, the Slovenian National Perinatal Information System maintained by the Slovenian National Institute of Public Health (http://www.nijz.si/); Sweden, the Swedish Medical Birth Register maintained by the Swedish National Board of Health and Welfare (www.socialstyrelsen.se) and complemented by linked data from Statistics Sweden (www.scb.se). All these data sources have been previously described [9–13].

As a comparator, we also included data from one state in the USA (California) using the vital statistics linked birth/death file from the California Department of Health Services (https://www.cdph.ca.gov/data/dataresources/requests/Pages/VitalStatisticsBirthDeathFetalDeathMarriageData.aspx) as previously described [14]. For all datasets ethics approval and access permissions were obtained where required (see S1 Appendix for more details).

### Definitions and preparation of the five individual patient-level datasets

Preterm birth and very preterm birth were defined as either spontaneous or provider-initiated deliveries occurring before 37 and 32 weeks of gestation, respectively. We classified preterm births into two general subtypes in order to explore how the effect of risk factors differs across these types: (1) spontaneous preterm birth (preterm labor or preterm prelabor rupture of fetal membranes before 37 weeks of gestation), and (2) provider-initiated preterm birth before 37 weeks of gestation, whether medically indicated or not [7, 15]. Gestational age was estimated using ultrasonography or in some cases menstrual history (see S1 Appendix for details for each dataset). We excluded stillbirths and multiple gestations from the analysis. Selection of preterm birth risk factors to be considered in the analysis was performed by a committee of 11 scientific experts (co-authors), based on their knowledge of existing risk factors as mentioned in the literature and the availability of data. Definitions of risk factors are provided in the S1 Appendix; any registry entry with missing or outlier data was excluded from the analysis (see S1 Appendix for exclusion criteria).

We further classified risk factors into 3 broad intervention areas which we termed, 1) research, 2) policy and public health, and 3) clinical practice. We did this for heuristic purposes, and made these distinctions based on our working group's expertise, as we were unable to find literature on any other group that had attempted to do this. The intervention area "research" comprises risk factors for which current biological or etiological understanding is insufficient to prevent preterm birth utilizing currently available clinical practice and/ or policy. Thus, more research is needed, specifically likely to involve cell biology or molecular underpinning responsible for association lacking an ostensible plausible explanation. "Policy and public health" interventions arises from risk factors whose prevalence could be impacted by policy and /or public health interventions and only to a limited extent by the actions of individual practitioners. "Clinical practice" interventions involve addressing risk factors whose prevalence could be directly impacted by clinicians adjusting their practice (e.g., elective preterm delivery). Some risk factors (e.g., diabetes mellitus and chronic hypertension) are relevant to both "policy and public health" and "clinical practice" and were thus considered into both categories.

### Regression models

**Fig 2:**
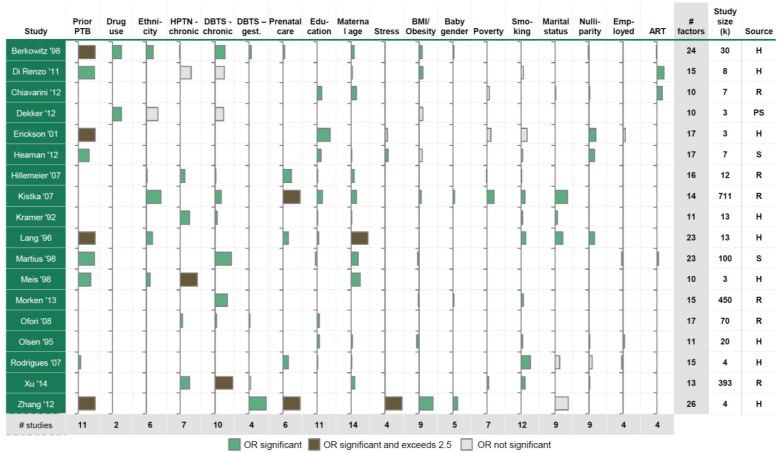
Systematic review of previously published multivariate analyses of preterm birth [19–36]. Each bar represents the difference between the reported odds ratios and 1; positive/negative bars are associated with increased or decreased risks of preterm birth respectively. Where categorical variables were reported, we reported the category having the largest significant odds ratio. We included all risk factors considered by at least 3 studies. Reference categories are the following: non-Hispanic white (ethnicity) (all studies, except Dekker '12 (non-Caucasian)); prenatal care beginning before 13 (Berkowitz '09) or 14 (Rodrigues '07) completed weeks of gestation, prenatal care received during first trimester (Lang '96), "adequate" (Hillemeier '07), received (Kristka '07), or not received (Zhang '12); high education (all, except Lang '96, Rodrigues '07), high-school graduate (Lang '96), 4–8 years of school education (Rodrigues '07); age > 20 (Kistka '07), age 20–29 (Berkowitz '09, Chiavarini '12), age 25–29 (Hillemeier '07), age 25–34 (Lang '96, Xu '14), age 18–30 (Meis '98), age 20–34 (Kramer '92), age 20–35 (Heaman '12, Olsen '95), age < 35 (Di Renzo '11); low perceived stress; healthy BMI; female baby; non-smokers; least deprived population; married. For categorical variables only the category with the largest significant odds ratio is shown; ethnicity: Black (Berkowitz '98, Hillemeier '07, Kristka '07, Meis '98, Lang '96), Caucasian (Dekker '12); education: lowest education (all studies); age: < 15 (Lang '96), < 20 (Berkowtiz '98, Kristka '07, Olsen '95), > 30 (Meis '98), > 35 (Di Renzo '11, Heaman '12, Hillemeier '07, Kramer '92, Rodrigues '07), > 40 (Chiavarini '12); BMI/Obesity: < 20 (Berkowtiz '98, Dekker '12, Kristka '07, Olsen '95), > 25 (Di Renzo '11), > 30 (Zhang '12), > 45 (Xu '14); poverty: high level (Erickson '01, Hillemeier '07, Kristka '07, Xu '14). A missing bar indicates that the risk factor was not considered in the study. Abbreviations: PTB, preterm birth; HPTN, hypertension; DBTS, diabetes; BMI, body mass index; gest., gestational; ART, assisted reproductive technology; OR, odds ratio; H, hospital; R, registry; S, survey; PS, prospective study.

In order to determine the independent contribution (i.e. after accounting for the impact of other factors) of each risk factor to an individual's risk for preterm birth, we created a model for each country including all risk factors. For analyses on the individual patient-level datasets (Figs 2 and 5 and Figs A, B, C of S1 Appendix), data were analyzed using SPSS 20.0 (IBM Corp.). We used logistic regression analysis to calculate odds ratios (ORs) and 95% confidence intervals (CIs) for each subtype of preterm birth. Full models were used that contained all appreciated preterm birth risk factors, with no stepwise regression. Sensitivity and specificity of logistic models were calculated as measures of the predictive capability of the models, i.e., the ability of the model to "predict" preterm birth [16]. Receiver operating characteristic (ROC) curves and area under curve (AUC) were also obtained to further assess predictive capability. Statistical significance of independent variables was defined as p < 0.05. The risk factor calculated p values for each logistic regression model are provided in S1 Appendix. Stratification analysis of spontaneous and provider-initiated preterm births was performed on three datasets where these data were available (New Zealand, Slovenia, and Sweden) (Figs A, B of S1 Appendix).

### Analysis of patient subpopulations with greatest risk of developing preterm births

To determine subpopulations with the greatest risk of developing preterm birth, we combined all four of the whole country datasets (Czech Republic, New Zealand, Slovenia, and Sweden) and the California state dataset to generate a total database of ~ 4.1 million singleton pregnancies. We then analyzed risk factors common to all five datasets. The risk factors were combined to define subpopulations each with a unique combination of preterm birth risk factors. Subpopulations with prevalence below 1 in 10,000 were excluded.

### Estimation of the impact of risk factors on singleton preterm birth rates

As reflected in Fig 5, we determined the absolute impact of each risk factor on preterm birth rates using the following equations to estimate the impact of each risk factor:

Prob(PTB| RF)≅Prob(RF)×AME(RF)

Where *Prob*(*PTB*|*RF*) is the probability of preterm birth given the risk factor RF, *Prob*(*RF*) is the prevalence of the risk factor RF, and *AME*(*RF*) is the average marginal effect of the risk factor RF. The latter is defined as

$$\text{AME}\left(\text{RF}\right)-\text{E}\left(Y_{1}-Y_{0}\right)$$

where *E()* indicates the expectation, *Y*_*1*_ is the outcome that would be obtained if a subject had the risk factor, and *Y*_*0*_ is the outcome that would be obtained if a subject had the risk factor [17, 18]. The prevalence was calculated from each dataset, and the average marginal effect was calculated using Stata 14.0 (StataCorp). The data in Fig 5 are displayed as "waterfall charts".

### Estimation of risk factor contributions to differences in overall preterm birth rates across countries

**Fig 3:**
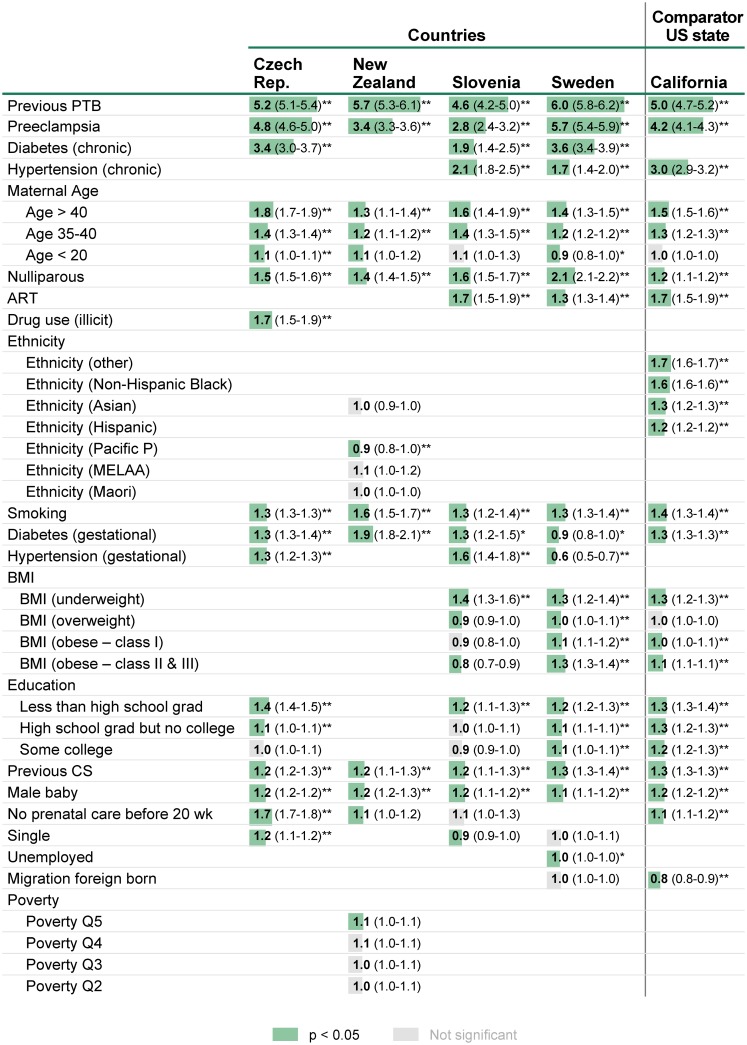
Risk factors for preterm birth across four countries and one comparator U.S. state. The odds ratios for each risk factor were calculated using five independent logistic regression models. Statistical significance was defined as p < 0.05. The width of the shaded lines are proportional to the reported odds ratios. A missing value indicates that data on risk factor were not available. For categorical variables, the reference categories were age 20–34 (age), non-Hispanic white (ethnicity), healthy BMI (BMI 18.5–24.9), highest education (college graduate or more), least deprived (poverty quintile Q1). Abbreviations: BMI, body mass index; MELAA, Middle Eastern, Latin American or African; PTB, preterm birth; ART, assisted reproductive technology; CS, cesarean section; 20wk, 20 weeks; Pacific P, Pacific people; * p<0.05; ** p<0.01

We illustrate in Fig 6 our estimated impact of each risk factor or clinical practice following methodology and assumptions described in detail in S1 Appendix. The majority of risk factors used previously (see Fig 3) were included in this comparison, as well as additional contributors including multiple pregnancy and preventive interventions (cervical cerclage, progesterone) not previously assessed. For some countries, we were unable to identify the prevalence of some risk factors which obviated our ability to estimate their contributions (marked as N/A in Fig 6). Briefly, for education, baby sex, access to maternity care, smoking, age, marital status, nulliparity, BMI, hypertension, diabetes, preeclampsia, and ART, we used a formula analogous to that applied in Fig 5 using the average AME calculated across the set of four countries and prevalence information from multiple sources (see S1 Appendix). For multifetal gestations, we summed the contributions of twins and triplets which were estimated by multiplying country-specific prevalence by the average preterm birth rates of twins and triplets both calculated from the set of four countries. For prior preterm birth and prior cesarean section, we estimated the prevalence of each risk factor using rates in Sweden, relative differences in preterm birth and parity, and average AMEs calculated across the set of four countries (see S1 Appendix for more details). For cervical cerclage and progesterone use, we used estimation methodologies and assumptions similar to those used in a previous study [5]. Data are presented in waterfall charts (as in Fig 6).

## Results

### Choosing Risk Factors

Systematic review of multivariate studies on preterm birth risk factors identified a total of 18 studies that considered a minimum of 6 non-clinical maternal or fetal risk factors as covariates (Fig 2 and "Research in context" panel) [19–36]. These studies, ranging from approximately 3000 up to 711,000 participants, were conducted in various geographies, including North America (Canada, USA), Europe (Denmark, Finland, Italy, Portugal, Sweden), Oceania (Australia, New Zealand), and Asia (China), on hospital populations, registry data, and surveys.

Most studies (11/18, 61%) were conducted within populations smaller than 15,000. Because data were generated in disparate locations and not subjected to a common template, the risk factors analyzed in each study were understandably variable both in number and types of risk factors collected. Very few studies (4/18, 22%) included more than 20 risk factors. For the 18 identified risk factors that were interrogated in at least 3 studies, reported odds ratios varied across studies up to 9-fold (Fig 2). At pregnancy inception the four factors most consistently associated with significantly increased risk of preterm birth were prior preterm birth, advanced maternal age, pregestational diabetes mellitus, and chronic hypertension. Inconsistent association (i.e. <50–75% of studies where studied) was observed with other factors including BMI, prenatal care, education, and poverty.

### Contribution of each risk factor to individual preterm birth risk

The contributions of each risk factor (or combination thereof) to individual preterm birth risk was estimated using logistic regression analysis of the large individual patient-level datasets from four countries (Czech Republic, New Zealand, Slovenia, Sweden), and compared these results to one heavily populated state from the United States with robust pregnancy data (California) (Fig 3). After exclusion of entries with outliers and/ or missing values, the total numbers of live singleton births and timeframes were: Czech Republic, 1,303K/2000-2013; New Zealand, 247K/2008-2012; Slovenia, 175K/2002-2012; Sweden, 1,086K/1998-2012; California, 1,339K/2008-2010. Overall, 21 risk factors for preterm birth were assessed in this cross-country analysis of 4.1 million live singleton births.

Odds ratios for each risk factor (Fig 3) were remarkably consistent across countries: 70% (17/24) of the odds ratios calculated over multiple geographies had maximum variation of less than 50%, whereas 87% (21/24) showed less than 100%. The two individual risk factors which were associated with the highest risk of preterm birth were prior preterm birth and preeclampsia (odds ratios 4.6–6.0 and 2.8–5.7 respectively). Other risk factors with association (odds ratios 1.2–3.6) with preterm birth include chronic diseases (pre-gestational diabetes and chronic hypertension), advanced maternal age (age > 40), ART, tobacco smoking, and illicit drug use; the p-values for all of these associations were <0.05. The effect of ethnicity could be studied in only two geographies where data were available. Significant effects were observed in the California dataset for several ethnic populations, most notably for Non-Hispanic Blacks (odds ratio 1.6). In contrast, ethnicity effects were small or nonexistent in New Zealand. Low BMI was associated with higher risk of preterm birth (odds ratios 1.3–1.4) whereas the effects of overweight or mild obesity (class I) were small, insignificant or inconsistent. With the exception of the lowest education level which was consistently associated with higher risk of preterm birth (odds ratios 1.2–1.4), other socioeconomic factors had little or no impact on preterm birth (odds ratios < 1.3). Consistent but small associations were observed for other risk factors including previous cesarean section, lack of prenatal care visit before 20 weeks (except Czech Republic), and male sex (odds ratio < 1.3). Despite relatively low odds ratios for nulliparity (1.4–2.1 in the four countries) or male sex (1.2 in 3 of the 4 countries), the high prevalence for these risk factors generated statistical significance for population attributable risks.

### Individual risk factors for spontaneous vs. provider-initiated preterm birth

In our stratification analysis, prior preterm birth remained by far the strongest risk factors for both provider-initiated and spontaneous preterm birth (odds ratios 4.5–7.1, Figs A, B of S1 Appendix). For provider-initiated preterm birth, major clinical risk factors (e.g., preeclampsia, chronic hypertension) ranked at the top (odds ratios 2.3–5.2) and other risk factors had much smaller or inconsistent contributions. For spontaneous preterm birth, chronic (pre-gestational) diabetes was very strongly associated with preterm birth (odds ratios 3.3–4.6). Nulliparity and prior cesarean birth were important risk factors for spontaneous (odds ratios 1.4–2.4) but not provider-initiated (odds ratios 0.5–1.6) preterm birth. The impact of other risk factors including ART, maternal age, and male sex, was consistently greater for spontaneous versus provider-initiated preterm birth. In the Swedish dataset, high BMI was a significant risk factor for spontaneous preterm birth (odds ratios 1.2–1.4) but was small or insignificant for provider-initiated preterm birth (odds ratios 0.9–1.0). In the Slovenian dataset, high BMI was insignificant for spontaneous preterm birth but protective for provider-initiated preterm birth (odds ratios 0.6–0.7).

### Individual risk factors for preterm birth vs. very preterm birth

The same risk factors were drivers for both preterm birth and very preterm birth (Fig C of S1 Appendix) although the magnitude of several risk factors (e.g., ethnicity, lower education, advanced age, and ART) was greater for very preterm birth. High BMI (obesity classes II-III) was strongly associated with very preterm birth in both the Swedish and California contexts (odds ratio 1.5–1.7) but not in the Slovenian context (odds ratio 0.8).

### Predictive performance of models

The predictive performance of all risk factors combined in logistic models was only modest. For example, Fig D of S1 Appendix shows the ROC curve for the Swedish analysis (see Fig 3) with an area under the curve of 0.662 and a sensitivity of 27% based on 90% specificity. Similar performance was achieved with the other datasets. Other predictive models were tested, including Decision Tree and Naive-Bayes classifiers, but did not show better predictive capabilities (data not shown).

**Fig 4:**
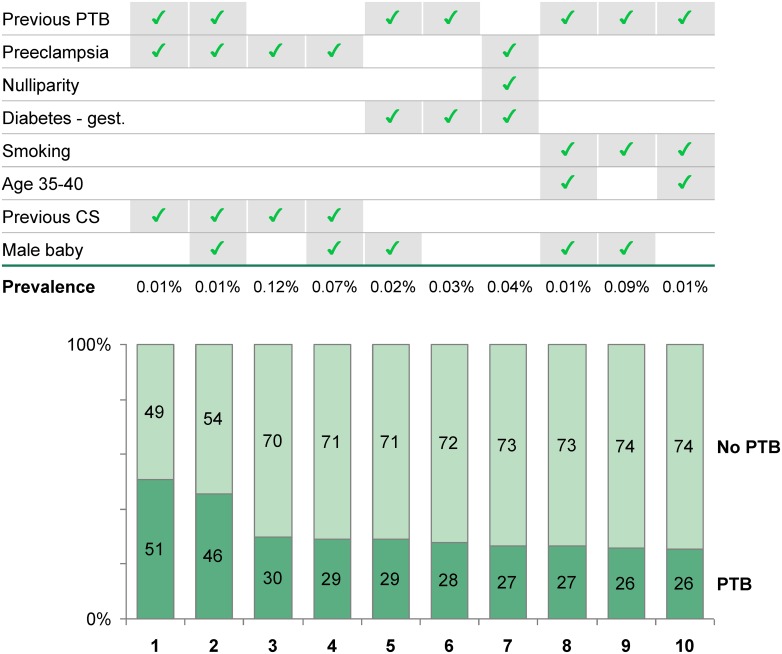
Top 10 subpopulations with highest probability of preterm birth. 4 datasets (New Zealand, Czech Republic, Slovenia, Sweden) representing a total of ~3 million singleton pregnancies were combined. Subpopulations were defined by their unique combinations of risk factors (top) and ranked by probability of preterm birth (bottom). This analysis was restricted to risk factors common to all four datasets. Subpopulations with prevalence below 1 in 10,000 were excluded. Additional subpopulations are shown in Fig E of S1 Appendix. Abbreviations: PTB, preterm birth; gest, gestational; CS, cesarean section.

Because predicting preterm birth is unsatisfactory using available data and tools, we leveraged the size and breadth of our datasets and analyzed patient subpopulations having the greatest risk to develop preterm birth. Strikingly, even for the 10 subpopulations with the strongest combinations of risk factors of preterm birth, the likelihood of a delivery at term was greater than 50% (Fig 4; additional subpopulations in Fig E of S1 Appendix).

### Impact on population risk for preterm birth

**Fig 5:**
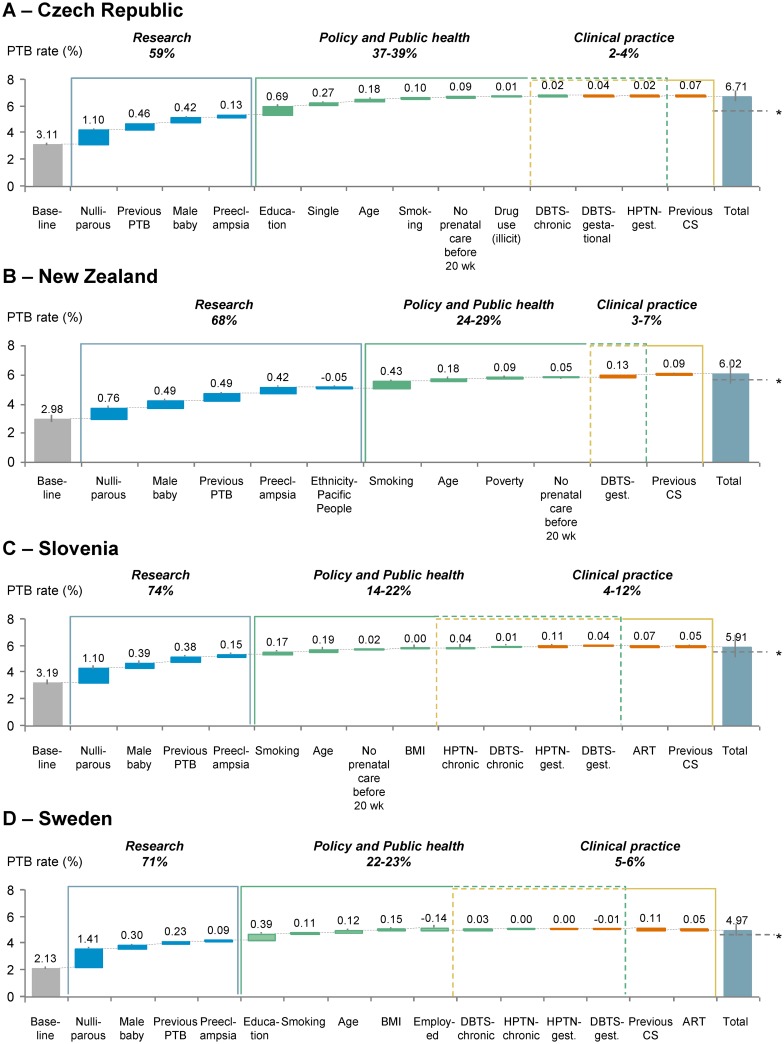
Estimated contribution of risk factors on population preterm birth rate and opportunities for various stratified interventions for (A) Czech Republic, (B) New Zealand, (C) Slovenia, (D) Sweden. Results from the logistic regression analysis were combined with prevalence of risk factor to estimate the impact of each risk factor (see Methods). Risk factors were grouped into three intervention areas, "Research", "Policy and Public Health", "Clinical Practice". Some risk factors (e.g. diabetes, hypertension) could and were classified into multiple categories. Percentage ranges indicated for "Policy and Public Health" and "Clinical Practice" thus reflect scenarios with or without inclusion of these overlapping risk factors. An asterisk represents the observed preterm birth rate in each dataset. Error bars: 95% confidence intervals. Abbreviations: PTB, preterm birth; HPTN, hypertension; DBTS, diabetes; CS, cesarean section; ART, assisted reproductive technology; 20wk, 20 weeks.

To estimate the contribution of individual risk factors for preterm birth on overall *population* preterm birth rates, we developed a model that combined odds ratios and prevalence of each risk factor (Fig 5). Risk factors were further classified into 3 broad intervention areas of research, policy / public health, and clinical practice (see methods). With this model, research constitute the most potentially impactful arena of intervention with the greatest estimated contribution to preterm birth (59–74%), followed by implementation of policy and public health actions (14–39%), and the least potential for impact with altered clinical practice (2–12%). Identifying the etiology of increased preterm birth risk associated with nulliparity and male sex within the research arena and eliminating this risk would contribute the most to the overall population preterm birth rates given the high prevalences of these risk factors (see Fig 3). Although preeclampsia is second only to prior preterm birth as an *individual* risk factor, it is significantly less impactful at the population level due to its relatively low prevalence (5–7%). Education, smoking, and maternal age constitute the main contributors to preterm birth amenable to policy and public health intervention. The baseline rates for lowest-risk populations (which were calculated from the intercepts of the logistic regression models) appear consistent across countries, ranging from 2.1% in Sweden to 3.2% in Slovenia. This rate reflects the risk for a pregnancy in which the mother is multiparous, has a female baby, has had no prior preterm birth, does not develop preeclampsia, is from the highest education group, andis characterized by lowest-risk categories.

### Explaining preterm birth variation across countries

We sought to estimate the relative contributions of identified risk factors to differences in preterm birth rate observed between Sweden (the country with the lowest preterm birth rate in our data set) and 15 other VHHDI countries (see S1 Appendix for data sources).

**Fig 6:**
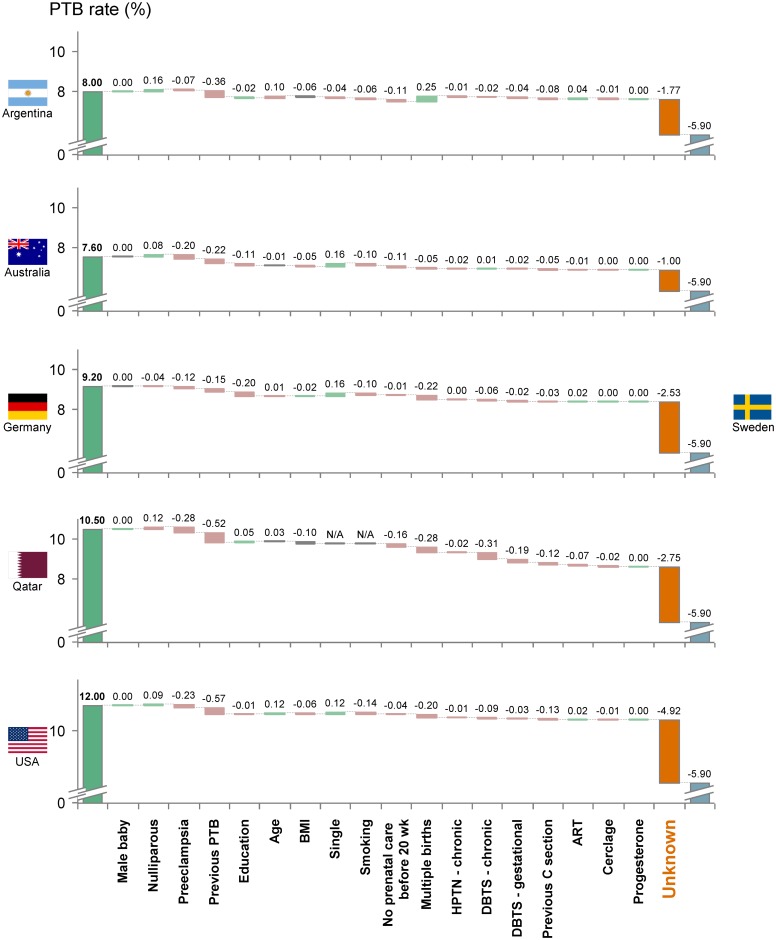
Estimated contributions of risk factors and clinical practices to differences in preterm birth rates between countries with VHHDI (left) and Sweden (right). The left and right bars represent the preterm birth rates for the indicated countries [5]. The size of each step in the “waterfall” was calculated by taking the difference in the estimated impact of risk factor (or clinical practice) between the indicated country and Sweden. The last step, labeled "unknown", represents the percentage not captured by the risk factors and clinical practices shown here. "N/A" indicates that information was not available to estimate the impact of the risk factor. Results for additional countries are shown in Fig F of S1 Appendix. Abbreviations: PTB, preterm birth; HPTN, hypertension; DBTS, diabetes; ART, assisted reproductive technology; 20 wk, 20 weeks.

The net contribution of the known set of preterm birth risk factors evaluated in this study is insufficient to explain the overall differences in preterm birth rates observed among other countries with VHHDI and Sweden (Fig 6 and Fig F of S1 Appendix). On average, all factors combined can explain only 37% of the difference across countries. For each country, the magnitude of the unexplained difference appears to be highly correlated (R^2^ = 0.96) to the overall difference in preterm birth rate between that country and Sweden, ranging from 0.1% (Japan) to 6.2% (Bahrain). Among all risk factors considered, preeclampsia, low education, multifetal gestation, previous preterm birth, and smoking are the main risk factors accounting for explainable differences among countries.

## Discussion

The unprecedented individual patient-level dataset (~5x larger than any used previously, and the first that is cross-country) and newly applied analytic approaches allowed us to make several intriguing advances with this study. First, we robustly confirmed prior preterm birth and preeclampsia as the top individual risk factors. We also robustly clarified the magnitude of impact of other risk factors, confirming some prior findings while not confirming others. Second, we identified nulliparity and male baby sex as the top contributors to the population preterm rate, and we found that unknown factors requiring further research to act upon account for ~2/3 of the preterm birth rate. Third, we found that the contribution of risk factors to preterm birth burden is remarkably similar across countries–both in terms of individual risk as well as population preterm rate. Fourth, we found that we are unable to account for ~2/3 of the difference in preterm birth rates between Sweden and 15 other countries with VHHDI. In addition to these intriguing advances, even though robust preterm birth prediction remains elusive, our analysis allows an improved capability to robustly assess preterm birth risk for an individual pregnant patient with a given set of risk factors.

We not surprisingly found previous preterm birth to be the strongest individual risk factor for both spontaneous and provider-initiated preterm birth (Figs A, B of S1 Appendix), consistent with a large body of prior published evidence and the known 25–30% heritability of preterm birth [19, 23, 24, 32, 37]. This risk factor likely reflects persistent genetic and epigenetic components, supplemented perhaps by an interaction with deleterious environmental risk factors which could not be captured by traditional variables either in our model or in other studies.

Preeclampsia was also confirmed as one of the top individual preterm birth risk factors, second only to prior preterm birth. Our study is novel, however, in that few other multivariate studies had been able to assess the variable of preeclampsia while controlling for other related confounding factors such as chronic hypertension.

Our strategy identified two risk factors with relatively low odds ratios but high prevalence, potentially exerting a strong if yet unexplained impact on population preterm birth rates due to their high prevalence. Nulliparity (prevalence 40–50%, odds ratios 1.2–2.1) appears to have the greatest single impact on population preterm birth rates across all risk factors considered, accounting for up to 28% of the total rate in Sweden. This impact is incremental to that driven by higher incidence of preeclampsia in nulliparous pregnancies, and could in part be due to the same unknown factors responsible for risk associated with recurrent preterm birth. Male sex has been previously identified as a risk factor for preterm birth [38]. Similar to nulliparity, we find in our very large sample that male sex (prevalence 51%, odds ratio 1.2 in 3 of 4 countries and California) contributes significantly to the population preterm birth rates (up to 8% in New Zealand) despite relatively small odds ratios. Based on our models, nulliparity accounts for 13–28% and male sex accounts for 6–8% of the population preterm birth rates within the four countries and the 2.8 million singleton births that were analyzed. Yet the biological explanation for these two risk factors remains unclear, and likewise the fashion in which this information could be used to reduce preterm birth is unclear.

Other significant risk factors, albeit with lower odds ratios (1.3–3.6, for all preterm births) confirmed through this analysis include chronic hypertension, pregestational diabetes mellitus, older maternal age, and ART, all holding for both spontaneous and provider-initiated preterm birth. Previous cesarean section is associated in our analysis with an increased risk of spontaneous preterm birth (odds ratios 1.4–1.9) but appears protective for provider-initiated preterm birth (odds ratios 0.5–0.9). This most likely reflects risks of early birth related to need for repeat cesarean delivery.

We confirmed robustly several other risk factors whose effects were not always consistent in every study using much smaller data sets or lacking multivariate analysis. Being underweight (BMI < 18.5 kg/m^2^) is a significant risk factor for both spontaneous and provider-initiated preterm birth (OR 1.3–1.4, p<0.01), whereas being overweight (BMI 25.0–29.9 kg/m^2^) or slightly obese (class I, BMI 30.0–34.9 kg/m^2^) are not (OR 0.9–1.1). Interestingly, being very obese (class II-III, BMI > 35 kg/m^2^) is a risk factor of preterm birth in Sweden and in California (odds ratios 1.1–1.3, p<0.001), as has been recently reported, but not in Slovenia [39]. This effect is greatly amplified for very preterm birth in both geographies (odds ratios 1.5–1.7). Also of great interest is our finding that being overweight or obese (class II-III) does not increase the risk of provider-initiated preterm birth (odds ratio 0.6–1.0), a finding that contradicts one recent report but confirms another [40, 41]. Our results likely are due to the benefit of our larger set of confounding factors that were available for adjustment. For example, variable and sometimes modest effects have been reported for tobacco smoking in prior studies (e.g. [19, 23]; however, we found a very robust effect across all five datasets considered (OR 1.3–1.6, p<0.001). Conflicting results have been reported on the impact of socioeconomic status on preterm birth (e.g. [24, 26]). Our analysis indicates a higher likelihood of preterm birth for patients having low education (high-school or less, odds ratios 1.2–1.4, p<0.001), an effect magnified for very preterm birth (odds ratios 1.3–1.8).

As expected, our results from the comparator U.S. state of California indicate an increased risk for preterm birth among Non-Hispanic Blacks (odds ratio 1.6). Inasmuch as we had ethnicity data only from New Zealand among the other countries analyzed, we are unable to fully explore the relationship of ethnicity to preterm birth that was evident in the California data. Since New Zealand’s patient population is fairly homogeneous compared to California’s very heterogenous population, we did not expect to find a relationship with ethnicity in that country nor might we in the relatively homogenous populations of Sweden, Slovenia or the Czech Republic. We also lacked sufficient information in the datasets to explore any possible association between migration and preterm birth.

Several investigators have recently addressed how differences in risk factor prevalence could explain variations in preterm birth rates across countries. A recent review summarized differences in preterm birth risk factors, reproductive health policy and medical practice, and measurement issues across European countries [42]. Zeitlin et al. [43] showed that a set of 7 key socio-demographic risk factors and differences in obstetric intervention are insufficient to explain the elevated preterm birth rate in the United States compared to France. A similar comparison of data from Canada and the United States reached the same conclusion [44]. Yet despite the inclusion of a larger set of risk factors associated with preterm birth, we were still able to explain only 37% of the differences in preterm birth rates among the 15 VHHDI countries considered and Sweden, the country having the lowest preterm birth rate (5.5%). Differences ranged from 13% in Singapore to 66% in France. Altogether the conclusions from our study confirm the paucity of factors which are amenable to provider-initiated intervention(s), as shown by Chang et al. [5].

Although we used very large validated datasets and multiple independent predictors, we were only able to achieve modest levels of combined specificity and sensitivity for the prediction of preterm birth (see Fig D of S1 Appendix). Two non-mutually exclusive reasons might explain these difficulties. First, there are doubtless additional key predictors not recognized or considered in our models such as regulatory genes, recent migration, and fetal growth indicators. Second, it would appear that birth at term could be a very robust process as shown by our subpopulation analysis (see Fig 4). Even pregnant women with the strongest combination of risk factors are still more likely to experience a birth at term than a preterm birth.

While we are unable to definitively predict preterm birth, our analysis does allow us robustly to estimate the risk of preterm birth across different subpopulations. For example, a woman with no risk factors (i.e. no prior preterm birth, no preeclampsia, multiparous, female baby, etc.) would have a preterm birth risk of <4%, and a woman with a prior preterm birth, preeclampsia, and prior c-section would have a 48% chance of a preterm birth. A further activity for this working group is to disseminate widely this capability to providers and potentially to patients. Although we do not yet know if this capability can be beneficial for clinical practice, certainly predictive capacity should be helpful as effective interventions become validated.

Our study is the first multi-country analysis to interrogate the impact of individual preterm birth risk factors within and across countries with VHHDI. High grade individual patient-level data from approximately 4.1 million singleton pregnancies were made available for analysis, including 2.8 million from 4 countries (New Zealand, Czech Republic, Slovenia, Sweden) and 1.3 million from a large comparator U.S. state (California). We were able to assess in various combinations the association of 21 individual risk factors with preterm birth, adjusting for multiple confounding effects. This unique dataset also enabled us to perform a first-of-its-kind analysis in preterm birth. We were able to look beyond simply individual risk to explore relative contributions of risk factors to population preterm birth rates.

The present investigation was limited to countries with VHHDI because of the scarcity of very high quality validated data collected in rigidly managed, monitored and nationally supported systems in low-income countries, even though such countries generally have higher rates of preterm births [2]. We have assumed our datasets are comparable in their measurement of key variables, including gestational age and others, which may not be the case. Additionally, we limited our analyses to a set of risk factors with available data and for which direct comparison across datasets was possible. Future studies will probe the association of other risk factors to preterm birth, including diet, stress, periodontal disease, and other maternal or fetal clinical risk factors, while adjusting for other confounding effects. The cross-country analysis undertaken was also limited to risk factors for which aggregate data were available for all countries considered. The effect of "unknown" contributors might be overestimated to some extent in these models.

In conclusion, our results strongly support the need for additional research to elucidate biological basis of many of the known associations with preterm birth. First, previous preterm birth is the top risk factor of preterm birth according to our logistic model, but as discussed above this risk factor is surely a surrogate for genetic, epigenetic and environmental causes not ostensibly captured among the risk factors for which we controlled. Likely these same causes are also responsible for the contribution of nulliparity to preterm birth. Second, classification of the risk factors into intervention areas highlights the importance for more etiologic research into the 65% of risk factors not susceptible to current policy, public health, or to clinical intervention (e.g. nulliparity, male baby sex). Third, the aggregated contribution of known risk factors is insufficient to explain differences in preterm birth rates observed between Sweden and 15 other countries with VHHDI. Overall, our results suggest that impactful progress in the prevention of preterm birth countries with VHHDI will only be made possible by research into the biology of human parturition.

## Supporting Information

S1 Appendix This file contains information on data access, ethical approvals, gestational age methodology, and variable definitions. It also includes the formulas and data sources used for the cross-country comparison as well as six supplementary figures and five supplementary tables.
(DOCX)

## References

[1] March of Dimes P, Save the Children, WHO. Born too soon: the global action report on preterm birth. 2012.

[2] Blencowe H, Cousens S, Oestergaard MZ, Chou D, Moller A-B, Narwal R, et al National, regional, and worldwide estimates of preterm birth rates in the year 2010 with time trends since 1990 for selected countries: a systematic analysis and implications. The Lancet. 2012;379(9832):2162–72. 10.1016/S0140-6736(12)60820-4 22682464

[3] Liu L, Oza S, Hogan D, Perin J, Rudan I, Lawn JE, et al Global, regional, and national causes of child mortality in 2000–13, with projections to inform post-2015 priorities: an updated systematic analysis. The Lancet. 2015;385(9966):430–40. 10.1016/S0140-6736(14)61698-6 25280870

[4] In: Behrman RE, Butler AS, editors. Preterm Birth: Causes, Consequences, and Prevention The National Academies Collection: Reports funded by National Institutes of Health. Washington (DC) 2007. 20669423

[5] Chang HH, Larson J, Blencowe H, Spong CY, Howson CP, Cairns-Smith S, et al Preventing preterm births: analysis of trends and potential reductions with interventions in 39 countries with very high human development index. Lancet. 2013;381(9862):223–34. 10.1016/S0140-6736(12)61856-X 23158883 PMC3572865

[6] Muglia LJ, Katz M. The enigma of spontaneous preterm birth. N Engl J Med. 2010;362(6):529–35. 10.1056/NEJMra0904308 20147718

[7] Blencowe H, Cousens S, Chou D, Oestergaard M, Say L, Moller AB, et al Born too soon: the global epidemiology of 15 million preterm births. Reprod Health. 2013;10 Suppl 1:S2 10.1186/1742-4755-10-S1-S2 24625129 PMC3828585

[8] Iams JD. Clinical practice. Prevention of preterm parturition. N Engl J Med. 2014;370(3):254–61. 10.1056/NEJMcp1103640 24428470

[9] Euro-Peristat. European Perinatal Health Report: The health and care of pregnant women and their babies in 2010. 2013.

[10] Lucovnik M, Tul N, Verdenik I, Blickstein I. Perinatal outcomes in singleton and twin pregnancies following first-trimester bleeding. J Perinatol. 2014;34(9):673–6. 10.1038/jp.2014.74 24786383

[11] Cnattingius S, Ericson A, Gunnarskog J, Kallen B. A quality study of a medical birth registry. Scand J Soc Med. 1990;18(2):143–8. 2367825 10.1177/140349489001800209

[12] A New Total Population Register System: More Possibilities and Better Quality Statistics Sweden 2002.

[13] Ministry of Health New Zealand Maternity Clinical Indicators 2012. 2014.

[14] Herrchen B, Gould JB, Nesbitt TS. Vital statistics linked birth/infant death and hospital discharge record linkage for epidemiological studies. Comput Biomed Res. 1997;30(4):290–305. 9339323 10.1006/cbmr.1997.1448

[15] Goldenberg RL, Gravett MG, Iams J, Papageorghiou AT, Waller SA, Kramer M, et al The preterm birth syndrome: issues to consider in creating a classification system. Am J Obstet Gynecol. 2012;206(2):113–8. 10.1016/j.ajog.2011.10.865 22177186

[16] Zweig MH, Campbell G. Receiver-operating characteristic (ROC) plots: a fundamental evaluation tool in clinical medicine. Clin Chem. 1993;39(4):561–77. 8472349

[17] Cole SR, Hernan MA. Constructing inverse probability weights for marginal structural models. Am J Epidemiol. 2008;168(6):656–64. 10.1093/aje/kwn164 18682488 PMC2732954

[18] VanderWeele TJ. Marginal structural models for the estimation of direct and indirect effects. Epidemiology. 2009;20(1):18–26. 10.1097/EDE.0b013e31818f69ce 19234398

[19] Berkowitz GS, Blackmore-Prince C, Lapinski RH, Savitz DA. Risk factors for preterm birth subtypes. Epidemiology. 1998;9(3):279–85. 9583419

[20] Di Renzo GC, Giardina I, Rosati A, Clerici G, Torricelli M, Petraglia F, et al Maternal risk factors for preterm birth: a country-based population analysis. Eur J Obstet Gynecol Reprod Biol. 2011;159(2):342–6. 10.1016/j.ejogrb.2011.09.024 22036591

[21] Chiavarini M, Bartolucci F, Gili A, Pieroni L, Minelli L. Effects of individual and social factors on preterm birth and low birth weight: empirical evidence from regional data in Italy. Int J Public Health. 2012;57(2):261–8. 10.1007/s00038-011-0311-3 22009490

[22] Dekker GA, Lee SY, North RA, McCowan LM, Simpson NA, Roberts CT. Risk factors for preterm birth in an international prospective cohort of nulliparous women. PLoS One. 2012;7(7):e39154 10.1371/journal.pone.0039154 22815699 PMC3398037

[23] Erickson K, Thorsen P, Chrousos G, Grigoriadis DE, Khongsaly O, McGregor J, et al Preterm birth: associated neuroendocrine, medical, and behavioral risk factors. J Clin Endocrinol Metab. 2001;86(6):2544–52. 11397853 10.1210/jcem.86.6.7607

[24] Heaman M, Kingston D, Chalmers B, Sauve R, Lee L, Young D. Risk factors for preterm birth and small-for-gestational-age births among Canadian women. Paediatr Perinat Epidemiol. 2013;27(1):54–61. 10.1111/ppe.12016 23215712

[25] Hillemeier MM, Weisman CS, Chase GA, Dyer AM. Individual and community predictors of preterm birth and low birthweight along the rural-urban continuum in central Pennsylvania. J Rural Health. 2007;23(1):42–8. 17300477 10.1111/j.1748-0361.2006.00066.x

[26] Kistka ZA, Palomar L, Lee KA, Boslaugh SE, Wangler MF, Cole FS, et al Racial disparity in the frequency of recurrence of preterm birth. Am J Obstet Gynecol. 2007;196(2):131 e1–6. 17306652 10.1016/j.ajog.2006.06.093

[27] Martius JA, Steck T, Oehler MK, Wulf KH. Risk factors associated with preterm (<37+0 weeks) and early preterm birth (<32+0 weeks): univariate and multivariate analysis of 106 345 singleton births from the 1994 statewide perinatal survey of Bavaria. Eur J Obstet Gynecol Reprod Biol. 1998;80(2):183–9. 9846665 10.1016/s0301-2115(98)00130-4

[28] Meis PJ, Goldenberg RL, Mercer BM, Iams JD, Moawad AH, Miodovnik M, et al The preterm prediction study: risk factors for indicated preterm births. Maternal-Fetal Medicine Units Network of the National Institute of Child Health and Human Development. Am J Obstet Gynecol. 1998;178(3):562–7. 9539527 10.1016/s0002-9378(98)70439-9

[29] Morken NH, Kallen K, Jacobsson B. Predicting risk of spontaneous preterm delivery in women with a singleton pregnancy. Paediatr Perinat Epidemiol. 2014;28(1):11–22. 10.1111/ppe.12087 24118026

[30] Ofori BD, Le Tiec M, Berard A. Risk factors associated with preterm birth according to gestational age at birth. Pharmacoepidemiol Drug Saf. 2008;17(6):556–64. 10.1002/pds.1575 18327870

[31] Olsen P, Laara E, Rantakallio P, Jarvelin MR, Sarpola A, Hartikainen AL. Epidemiology of preterm delivery in two birth cohorts with an interval of 20 years. Am J Epidemiol. 1995;142(11):1184–93. 7485065 10.1093/oxfordjournals.aje.a117577

[32] Zhang YP, Liu XH, Gao SH, Wang JM, Gu YS, Zhang JY, et al Risk factors for preterm birth in five Maternal and Child Health hospitals in Beijing. PLoS One. 2012;7(12):e52780 10.1371/journal.pone.0052780 23300774 PMC3531336

[33] Xu XK, Wang YA, Li Z, Lui K, Sullivan EA. Risk factors associated with preterm birth among singletons following assisted reproductive technology in Australia 2007-2009-a population-based retrospective study. BMC Pregnancy Childbirth. 2014;14(1):406. 25481117 10.1186/s12884-014-0406-y PMC4266897

[34] Rodrigues T, Barros H. Comparison of risk factors for small-for-gestational-age and preterm in a Portuguese cohort of newborns. Matern Child Health J. 2007;11(5):417–24. 17342421 10.1007/s10995-007-0195-2

[35] Kramer MS, McLean FH, Eason EL, Usher RH. Maternal nutrition and spontaneous preterm birth. Am J Epidemiol. 1992;136(5):574–83. 1442721 10.1093/oxfordjournals.aje.a116535

[36] Lang JM, Lieberman E, Cohen A. A comparison of risk factors for preterm labor and term small-for-gestational-age birth. Epidemiology. 1996;7(4):369–76. 8793362 10.1097/00001648-199607000-00006

[37] Kistka ZA, DeFranco EA, Ligthart L, Willemsen G, Plunkett J, Muglia LJ, et al Heritability of parturition timing: an extended twin design analysis. Am J Obstet Gynecol. 2008;199(1):43 e1–5. 18295169 10.1016/j.ajog.2007.12.014

[38] Di Renzo GC, Rosati A, Sarti RD, Cruciani L, Cutuli AM. Does fetal sex affect pregnancy outcome? Gend Med. 2007;4(1):19–30. 17584623 10.1016/s1550-8579(07)80004-0

[39] Shaw GM, Wise PH, Mayo J, Carmichael SL, Ley C, Lyell DJ, et al Maternal prepregnancy body mass index and risk of spontaneous preterm birth. Paediatr Perinat Epidemiol. 2014;28(4):302–11. 10.1111/ppe.12125 24810721

[40] Cnattingius S, Villamor E, Johansson S, Edstedt Bonamy AK, Persson M, Wikstrom AK, et al Maternal obesity and risk of preterm delivery. JAMA. 2013;309(22):2362–70. 10.1001/jama.2013.6295 23757084

[41] Gould JB, Mayo J, Shaw GM, Stevenson DK, March of Dimes Prematurity Research Center at Stanford University School of M. Swedish and American studies show that initiatives to decrease maternal obesity could play a key role in reducing preterm birth. Acta Paediatr. 2014;103(6):586–91. 10.1111/apa.12616 24575829

[42] Delnord M, Blondel B, Zeitlin J. What contributes to disparities in the preterm birth rate in European countries? Curr Opin Obstet Gynecol. 2015;27(2):133–42. 10.1097/GCO.0000000000000156 25692506 PMC4352070

[43] Zeitlin J, Blondel B, Ananth CV. Characteristics of Childbearing Women, Obstetrical Interventions and Preterm Delivery: A Comparison of the US and France. Matern Child Health J. 2015;19(5):1107–14. 10.1007/s10995-014-1602-0 25119892

[44] Garn JV, Nagulesapillai T, Metcalfe A, Tough S, Kramer MR. International comparison of common risk factors of preterm birth between the U.S. and Canada, using PRAMS and MES (2005–2006). Matern Child Health J. 2015;19(4):811–8. 10.1007/s10995-014-1576-y 25060811 PMC4357269

