# Supplementary material for: Cross-Country Individual Participant Analysis of 4.1 Million Singleton Births in 5 Countries with Very High Human Development Index Confirms Known Associations but Provides No Biologic Explanation for 2/3 of All Preterm Births
Source: PLoS One. 2016 Sep 13;11(9):e0162506. doi: 10.1371/journal.pone.0162506 (PMC5021369; doi:10.1371/journal.pone.0162506)
Supplement: S1 Appendix — It also includes the formulas and data sources used for the cross-country comparison as well as six supplementary figures and five supplementary tables. (DOCX) [file pone.0162506.s001.docx]

**S1 Appendix**

**Cross-country individual participant analysis of 4.1 million singleton births in 5 countries with very high human development index confirms known associations but provides no biologic explanation for 2/3 of all preterm births**

David M. Ferrero, Jim Larson, Bo Jacobsson, Gian Carlo Di Renzo, Jane E. Norman, James N. Martin Jr, Mary D’Alton, Ernesto Castelazo, Chris P. Howson, Verena Sengpiel, Matteo Bottai, Jonathan A. Mayo, Gary M. Shaw, Nataša Tul, Petr Velebil, Sarah Cairns-Smith, Hamid Rushwan, Sabaratnam Arulkumaran, Jennifer L. Howse, Joe Leigh Simpson

Contents

[1 Data access, ethical approvals, and gestational age methodology 3](#_Toc460062792)

[2 Variable definitions and reference groups 5](#_Toc460062793)

[3 Formulas and data sources used for cross-country comparison 7](#_Toc460062794)

[3.1 Education, male sex, prenatal care, smoking, maternal age, marital status, nulliparity, BMI, chronic hypertension, chronic diabetes, preeclampsia, and ART 7](#_Toc460062795)

[3.2 Multiple gestations 10](#_Toc460062796)

[3.3 Previous preterm birth 10](#_Toc460062797)

[3.4 Previous cesarean section 11](#_Toc460062798)

[3.5 Cervical cerclage 11](#_Toc460062799)

[3.6 Progesterone use 12](#_Toc460062800)

[4 Supplementary Figures and Data 13](#_Toc460062801)

[4.1 Figs A, B, C 13](#_Toc460062802)

[4.1.1 Legend 13](#_Toc460062803)

[4.1.2 Fig A – spontaneous preterm birth 14](#_Toc460062804)

[4.1.3 Fig B – provider-initiated preterm birth 15](#_Toc460062805)

[4.1.4 Fig C – very preterm birth 16](#_Toc460062806)

[4.2 Fig D 17](#_Toc460062807)

[4.3 Fig E 18](#_Toc460062808)

[4.4 Fig F 21](#_Toc460062809)

[4.5 Supplementary Tables 24](#_Toc460062810)

[4.5.1 Legend 24](#_Toc460062811)

[4.5.2 Odds ratios and significance (Fig 3) 25](#_Toc460062812)

[4.5.3 Odds ratios and significance (Fig A of S1 Appendix) 26](#_Toc460062813)

[4.5.4 Odds ratios and significance (Fig B of S1 Appendix) 27](#_Toc460062814)

[4.5.5 Odds ratios and significance (Fig C of S1 Appendix) 28](#_Toc460062815)

[4.5.6 95% confidence intervals (Fig 3) 29](#_Toc460062816)

[5 Additional references 30](#_Toc460062817)

# Data access, ethical approvals, and gestational age methodology

*Czech Republic*: The Institute of the Health Information and Statistics of the Czech Republic approved the use of data from the Czech Republic and National Register of Mothers at Childbirth (NRROD) and National Register of Newborns (NRNAR).

- Gestational age methodology: defined as the difference between date of birth and date of last menstrual period (in completed weeks)

*New Zealand*: The Ministry of Health of New Zealand approved the use of data from the National Maternity Collection (MAT).

- Gestational age methodology: calculated from the date of the first day of a woman’s last menstrual period and her infant's date of birth, or derived from clinical assessment during pregnancy, or derived from an examination of the infant after birth

*Slovenia*: The National Institute of Public Health of Slovenia (NIJZ) approved the use of data from the Slovenian Perinatal Registry (NPIS).

- Gestational age methodology: Gestational age is given by three means: LMP, ultrasound (US) assessment in first half of pregnancy and pediatrician assessment of baby after birth. Algorithm for determining gestational age is as follows: in general, LMP is used. In cases where LPM is unknown or the difference between LMP and US is more than 7 days, US estimation is used. In rare cases when difference between GA estimated my LMP or US in pregnancy and pediatrician is greater than 2 weeks, pediatrician assessment is considered.

*Sweden*: The ethics committee, Göteborg, Sweden, approved the study (Reference number: Göteborg dnr 968-14). The National Board of Health and Welfare and Statistics Sweden approved the use of data from the Swedish Medical Birth Register with linkage to data from Statistics Sweden.

- Gestational age methodology: In the Swedish Medical Birth Register, the following sources are available to estimate gestational age: date of last menstrual period, corrected expected date of parturition according to last menstrual period (the weighted estimate made by the midwife at the antenatal care center basically based on last menstrual period and the length of the menstrual cycle), expected date of parturition according to ultrasound, and the estimated gestational age at birth reported by the delivery unit. Using these sources in a hierarchical arrangement, the best available estimate of gestational age for each infant is determined and designated ‘best estimate’. According to this method, the gestational age according to ultrasound was preferred when available and not too incongruous with the other sources.

*California*:

- Gestational age methodology: The best obstetric estimate of the infant’s gestation in completed weeks based on the birth attendant’s final estimate of gestation. This estimate of gestation should be determined by all perinatal factors and assessments such as ultrasound, but not the neonatal exam. Ultrasound taken early in pregnancy is preferred. (From National Vital Statistics System / Centers for Disease Control and Prevention guide)

# Variable definitions and reference groups

| **Master variable** | **Variable type** | **Inclusion/ Exclusion criteria** | **Description** |
| --- | --- | --- | --- |
|  |  |  |  |
| **Ethnicity** | Nominal |  | One category per ethnic group; reference: White Europeans |
| **Migration** | Binary |  | 1st generation migrant or not; reference: not 1st generation migrant |
| **Education** | Ordinal |  | 4 categories: 1) less than high school graduate, 2) high school graduate (or equivalent) but not college, 3) some college, 4) college graduate or more; reference: college graduate or more |
| **Poverty** | Ordinal |  | 5 deprivation quintiles: Q1 (least deprived) to Q5 (most deprived); reference: Q1 |
| **Mother employed** | Binary |  | Employment (full-time or part-time) of mother; reference: employed |
| **Marital status** | Binary |  | Marital status of mother; reference: married |
| **Mother age** | Ordinal | Include age 13-59 | 4 age categories with relevance for preterm birth: 1) < 20, 2) 20-34, 3) 35-39, 4) > 40 years old; reference: 20-34 years old |
| **Obesity at first visit** | Ordinal |  | 5 categories following WHO classification (1): 1) < 18.5 (underweight), 2) 18.5-24.9 (healthy), 3) 25-29.9 (overweight), 4) 30-34.9 (obese – class I), 5) > 35 kg/m^2^ (obese – class II & III); reference: healthy BMI |
| **Hypertension - gestational** | Binary |  | Reference: no gestational hypertension |
| **Hypertension - chronic** | Binary |  | Reference: no chronic hypertension prior to pregnancy |
| **Preeclampsia** | Binary |  | Reference: no preeclampsia |
| **Diabetes – gestational** | Binary |  | Reference: no gestational diabetes |
| **Diabetes - chronic** | Binary |  | Reference: no chronic diabetes |
| **Smoking** | Binary |  | Mother smoking at first prenatal care visit; reference: not smoking |
| **Drug use (illicit)** | Binary |  | Illicit drug use at first visit; reference: no illicit drug use |
| **ART** | Binary |  | Pregnancy resulting from use of artificial reproductive technology |
| **Prenatal care** | Binary |  | Timing of first prenatal care visit; reference: mother had first prenatal care visit before 20 weeks of gestation |
| **Baby sex** | Binary |  | Reference: female baby sex |
| **Prior C section** | Binary |  | Reference: mother did not undergo prior caesarean section |
| **Parity** | Binary | Exclude if  parity > 13 | Reference: non-nulliparous |
| **Preterm flag** | Binary | Include 20-44 weeks of gestation | Birth occurring before 37 weeks of gestation |
| **Very preterm flag** | Binary | Include 20-44 weeks of gestation | Birth occurring before 32 weeks of gestation |
| **Prior preterm birth** | Binary |  | Reference: no prior preterm birth |

# Formulas and data sources used for cross-country comparison

## Education, male sex, prenatal care, smoking, maternal age, marital status, nulliparity, BMI, chronic hypertension, chronic diabetes, preeclampsia, and ART

- The following formula was used to calculate the difference in contribution between each of the 15 VHHDI countries ("Country 1") and Sweden:

$$\left( Risk Factor {Prevalence}_{Country 1}\times{Risk Factor AME}_{Average} \right)-\left( Risk Factor {Prevalence}_{Sweden}\times{Risk Factor AME}_{Sweden} \right)$$

Where$Risk Factor Prevalence$ is the prevalence of the risk factor in Country 1 or Sweden as indicated, ${Risk Factor AME}_{Average}$ is the average marginal effect of the risk factor averaged across the four country datasets, and ${Risk Factor AME}_{Sweden}$ is the average marginal effect of the risk factor calculated from the Swedish dataset.

- For *prenatal care*, we estimated the percentage of pregnant women with first prenatal care visit after 20 weeks of gestation from a linear regression using antenatal care coverage by country (at least four prenatal care visits) and the percentages of pregnant women with first prenatal care visit after 20 weeks of gestation calculated from the four country datasets (R^2^ = 0.73). The same approach was used to estimate *nulliparity* using parity data available for all countries (R^2^ = 0.69). For *maternal age*, prevalence was estimated for each age group (< 20, 20-35, 35-40, > 40 years old) separately by multiplying the specific fertility rate and population by age group, and dividing this product by its sum across all age groups; the summed contribution from all age groups was reported in the figures (see Fig 6, Fig F of S1 Appendix). *Chronic hypertension* prevalence was estimated by weighting the female hypertension prevalence of each age group by their fertility rate. *Chronic diabetes* prevalence was estimated by multiplying the prevalence of chronic diabetes among pregnant women in Sweden by the ratio of chronic diabetes prevalence between Country 1 and Sweden.
- The prevalence of risk factors were obtained or inferred from the following sources:
  - *Education*: UNESCO, Institute for Statistics (2001-2012)
  - *Male sex*: United Nations, Department of Economic and Social Affairs, Population Division (2013)
  - *Prenatal care*: UNESCO, Global Databases, Antenatal care coverage - at least four visits (2007-2012); WHO, Global Health Observatory data, Antenatal care (at least 4 visits) (2007-2012)
  - *Smoking*: Argentina (2), Australia (Australian Institute of Health and Welfare), Canada (Network for the Prevention of Gestational and Neonatal Exposure to Tobacco Smoke, Center for Addiction and Mental Health, University of Toronto, Ontario), Chile (3), Czech Republic (dataset), France (4), Germany (5), Japan (3), New Zealand (dataset), Slovenia (dataset), Sweden (dataset), United Kingdom (Health & Social Care Information Center, 2013), USA (US Dept. of Health and Human Services, Results from the 2013 National Survey on Drug Use and Health: Summary of National Findings); data for Bahrain, Qatar, and Singapore were not available.
  - *Maternal age*: specific fertility rates and population data were obtained from the United Nations (World Fertility Data, 2012; World Population Prospects: The 2012 Revision)
  - *Marital status*: Child Trends (World Family Map 2013), except for Czech Republic and Slovenia (Eurostat data, Share of live births outside marriage). Data for Qatar, Bahrain, and Singapore were not available.
  - *Nulliparity*: data on births per woman from UNESCO, Institute for Statistics (2011)
  - *BMI - obese*: Global Health Observatory Data Repository, WHO (2008)
  - *BMI – underweight*: The WHO Global Database on Body Mass Index was used for all countries, except Argentina (UN Millennium Development Goals Report, 2014), Canada (Statistics Canada, 2011), Germany (6), Japan (Ministry of Health, Labour and Welfare), United Kingdom (National Obesity Observatory report on adult weight, 2011), USA (Results from the 2007-2008 National Health and Nutrition Examination Survey, CDC, National Center for Health Statistics), Bahrain (7), Singapore (OECD Statistics, Asia/Pacific 2012). Data for Qatar were not available.
  - *Chronic hypertension*: Fertility rates obtained from UNESCO, Institute for Statistics (2011); hypertension prevalence: Argentina (8) , Canada (Statistics Canada), Germany (9), Japan (10), United Kingdom (Health Survey for England, Health & Social Care Information Center, 2012), USA (National Center for Health Statistics - Health, United States, 2013), Qatar (11), Slovenia (report from The National Institute of Public Health of Slovenia, 2012), Czech Republic (12), New Zealand (The Health of New Zealand Adults 2011/12, Ministry of Health of New Zealand), Sweden (13), Australia (Australian heart disease statistics 2014, Heart Foundation), Singapore (National Health Survey 2010, Ministry of Health)
  - *Chronic diabetes*: prevalence for the 20-39 year old age group was obtained from the Diabetes Atlas (International Diabetes Federation, 2014)
  - *Preeclampsia*: Argentina (WHO - Global survey on maternal and perinatal health, 2004-2008), Australia (14), Canada (14), Czech Republic (dataset), France (15), Germany (16), Japan (WHO - Global survey on maternal and perinatal health, 2004-2008), New Zealand (dataset), Qatar (17), Singapore (18), Slovenia (dataset), Sweden (dataset), United Kingdom (NHS preeclampsia portal), United States (19). Data for Bahrain and Chile were not available.
  - *ART*: (20). Data for Bahrain and Singapore were not available.

## Multiple gestations

- The following formula was used:

$$\left[ \left( Twin prevalence \times Twin PTB rate \right)+\left( Triplet prevalence \times Triplet PTB rate \right) \right]_{Country 1}-\left[ \left( Twin prevalence \times Twin PTB rate \right)+\left( Triplet prevalence \times Triplet PTB rate \right) \right]_{Sweden}$$

Where $Twin PTB \mathrm{rate}$ and $Triplet PTB rate$ are the average rates of preterm birth for twins and triplets calculated from the four country datasets.

- Twin and triplet prevalence data was obtained from the United Nations Statistics Division (2007-2013)

## Previous preterm birth

- The following formula was used:

$$\left[ {Previous PTB Prevalence}_{Sweden}\times\frac{{PTB rate}_{Country 1}}{{PTB rate}_{Sweden}}\times\frac{{Parity}_{Country 1}}{{Parity}_{Sweden}}\times{AME}_{Previous PTB-Average} \right]-\left[ {Previous PTB Prevalence}_{Sweden}\times{AME}_{Previous PTB- Sweden} \right]$$

Where ${Previous PTB Prevalence}_{Sweden}$ is the prevalence of preterm birth in Sweden, $PTB rate$ is the rate of preterm birth in Country 1 or Sweden as indicated, $Parity$ is the average parity in Country 1 or Sweden as indicated, and ${AME}_{Previous PTB}$ is the average marginal effect of previous preterm birth averaged across the four country datasets or calculated from the Swedish dataset as indicated.

- Prevalence of preterm birth in Sweden was calculated from the dataset; preterm birth rates were obtained from (3) and parity data from the UNESCO Institute for Statistics (2011)

## Previous cesarean section

- The following formula was used:

$$\left[ {Previous CS Prevalence}_{Sweden}\times\frac{{PTB rate}_{Country 1}}{{PTB rate}_{Sweden}}\times\frac{{Parity}_{Country 1}}{{Parity}_{Sweden}}\times{AME}_{Previous CS -Average} \right]-\left[ {Previous CS Prevalence}_{Sweden}\times{AME}_{Previous CS - Sweden} \right]$$

Where ${Previous CS Prevalence}_{Sweden}$ is the prevalence of previous cesarean section in Sweden, $PTB rate$ is the rate of preterm birth in Country 1 or Sweden as indicated, $Parity$ is the average parity in Country 1 or Sweden as indicated, and ${AME}_{Previous CS}$ is the average marginal effect of previous cesarean section averaged across the four country datasets or calculated from the Swedish dataset as indicated.

- Prevalence of previous cesarean section in Sweden was calculated from the dataset; preterm birth rates were obtained from (3) and parity data from the UNESCO Institute for Statistics (2011)

## Cervical cerclage

- The following formula was used:

$$\left[ {{PTB attributed to prior PTB without SC}_{Country 1}-PTB attributed to prior PTB without SC}_{Sweden} \right] \times Cerclage prevalence \times Reduction of PTB with cerclage$$

Where ${PTB attributed to prior PTB without SC}$is the estimated percentage of preterm birth attributed to prior preterm birth for women without short cervix (3), $Cerclage prevalence$ is the prevalence of cervical cerclage (3), and $Reduction of PTB with cerclage$ is the estimated efficacy of cerclage to reduce preterm birth (3). Both the prevalence and efficacy of cerclage were modeled as global and the same estimations as Chang et al. (3) were used.

## Progesterone use

- The following formula was used:

$$\left[ {{PTB attributed to prior PTB without SC}_{Country 1}-PTB attributed to prior PTB without SC}_{Sweden} \right] \times Progesterone prevalence \times Reduction of PTB with progesterone$$

Where ${PTB attributed to prior PTB without SC}$is the estimated percentage of preterm birth attributed to prior preterm birth for women without short cervix (3), $Progesterone prevalence$ is the prevalence of progesterone use (3), and $Reduction of PTB with progesterone$ is the estimated efficacy of progesterone use to reduce preterm birth (3). Both the prevalence and efficacy of progesterone use were modeled as global and the same estimations as Chang et al. (3) were used.

# Supplementary Figures and Data

## Figs A, B, C

### Legend

Risk factors for **(A)** spontaneous, **(B)** provider-initiated preterm birth, and (**C**) very preterm birth. The odds ratios were calculated using independent logistic regression models for each country. Statistical significance was defined as p < 0.05. A missing value indicates that data on the risk factor was not available. For categorical variables, the reference categories were the following: age 20-34 (age), non-Hispanic white (ethnicity), healthy BMI (BMI 18.5-24.9), highest education (college graduate or more), least deprived (poverty quintile Q1). Abbreviations: PTB, preterm birth; ART, assisted reproductive technology; CS, caesarean section; 20wk, 20 weeks.

### Fig A – spontaneous preterm birth

### Fig B – provider-initiated preterm birth

### Fig C – very preterm birth

## Fig D

Representative receiver operating characteristic (ROC) curve and "area under curve" for one logistic model. This example is derived from the logistic model for preterm birth calculated from the Swedish dataset.

## Fig E

58 subpopulations with highest probability of preterm birth. 4 datasets (New Zealand, Czech Republic, Slovenia, Sweden) representing a total of ~ 3 million singleton pregnancies were combined. Subpopulations were defined by their unique combinations of risk factors (table) and ranked by probability of preterm birth (graph and last column of table). This analysis was restricted to risk factors common to all four datasets. Subpopulations with prevalence below 1 in 10,000 were excluded.


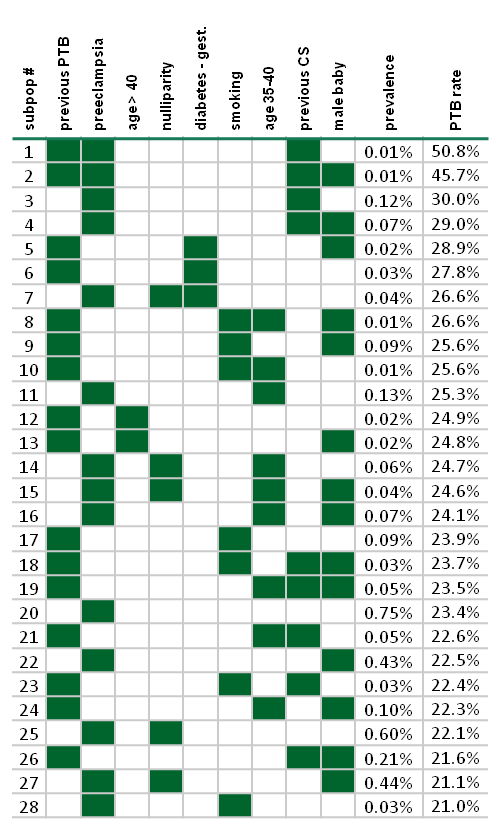

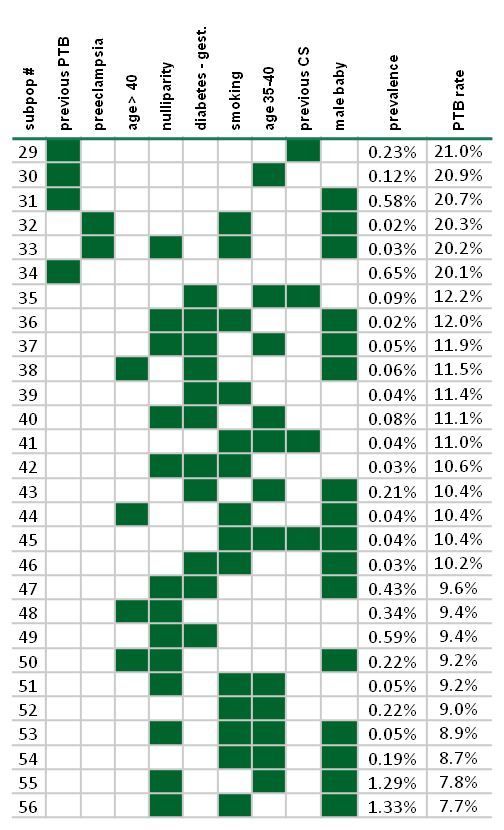


## Fig F

Estimated contributions of risk factors and clinical practices to differences in preterm birth rates between countries with VHHDI (left) and Sweden (right). The left and right bars represent the preterm birth rates for the indicated countries (3). The size of each step in the waterfall was calculated by subtracting the estimated impact of risk factor (or clinical practice) for the indicated country and Sweden. The last step, labeled "unknown", represents the percentage not captured by the risk factors and clinical practices interrogated. "N/A" indicates that no information was available to estimate the impact of that risk factor. Abbreviations: PTB, preterm birth; HPTN, hypertension; DBTS, diabetes; ART, assisted reproductive technology; 20 wk, 20 weeks.

## Supplementary Tables

### Legend

Odds ratios and significance (p value) of risk factors considered in Fig 2 and Figs A, B, C of S1 Appendix. The first number is the odds ratio; the number in parenthesis is the p value for p > 0.05. *p < 0.05; **p < 0.01; ***p < 0.001.

### Odds ratios and significance (Fig 3)

|  | **Czech Republic** | **New Zealand** | **Slovenia** | **Sweden** | **California** |
| --- | --- | --- | --- | --- | --- |
| Previous PTB | 5.2*** | 5.7*** | 4.6*** | 6*** | 5*** |
| Preeclampsia | 4.8*** | 3.4*** | 2.8*** | 5.7*** | 4.2*** |
| Diabetes (chronic) | 3.4*** |  | 1.9*** | 3.6*** |  |
| Hypertension (chronic) |  |  | 2.1*** | 1.7*** | 3*** |
| Maternal Age |  |  |  |  |  |
| Age > 40 | 1.8*** | 1.3*** | 1.6*** | 1.4*** | 1.5*** |
| Age 35-40 | 1.4*** | 1.2*** | 1.4*** | 1.2*** | 1.3*** |
| Age < 20 | 1.1*** | 1.1* | 1.1 (0.167) | 0.9** | 1 (0.313) |
| Nulliparous | 1.5*** | 1.4*** | 1.6*** | 2.1*** | 1.2*** |
| ART |  |  | 1.7*** | 1.3*** | 1.7*** |
| Drug use (illicit) | 1.7*** |  |  |  |  |
| Ethnicity |  |  |  |  |  |
| Ethnicity (other) |  |  |  |  | 1.7*** |
| Ethnicity (Non-Hispanic Black) |  |  |  |  | 1.6*** |
| Ethnicity (Asian) |  | 1 (0.440) |  |  | 1.3*** |
| Ethnicity (Hispanic) |  |  |  |  | 1.2*** |
| Ethnicity (Pacific P) |  | 0.9*** |  |  |  |
| Ethnicity (MELAA) |  | 1.1 (0.220) |  |  |  |
| Ethnicity (Maori) |  | 1 (0.763) |  |  |  |
| Smoking | 1.3*** | 1.6*** | 1.3*** | 1.3*** | 1.4*** |
| Diabetes (gestational) | 1.3*** | 1.9*** | 1.3** | 0.9** | 1.3*** |
| Hypertension (gestational) | 1.3*** |  | 1.6*** | 0.6*** |  |
| BMI |  |  |  |  |  |
| BMI (underweight) |  |  | 1.4*** | 1.3*** | 1.3*** |
| BMI (overweight) |  |  | 0.9 ** | 1*** | 1 (0.087) |
| BMI (obese – class I) |  |  | 0.9 (0.068) | 1.1*** | 1*** |
| BMI (obese – class II & III) |  |  | 0.8 *** | 1.3*** | 1.1*** |
| Education |  |  |  |  |  |
| Less than high school grad | 1.4*** |  | 1.2*** | 1.2*** | 1.3*** |
| High school grad but no college | 1.1*** |  | 1 (0.701) | 1.1*** | 1.3*** |
| Some college | 1 (0.055) |  | 0.9 (0.192) | 1.1*** | 1.2*** |
| Previous CS | 1.2*** | 1.2*** | 1.2*** | 1.3*** | 1.3*** |
| Male baby | 1.2*** | 1.2*** | 1.2*** | 1.1*** | 1.2*** |
| No prenatal care before 20 wk | 1.7*** | 1.1* | 1.1 (0.128) |  | 1.1*** |
| Single | 1.2*** |  | 0.9* | 1 (0.514) |  |
| Unemployed |  |  |  | 1** |  |
| Migration foreign born |  |  |  | 1 (0.178) | 0.8*** |
| Poverty |  |  |  |  |  |
| Poverty Q5 |  | 1.1* |  |  |  |
| Poverty Q4 |  | 1.1 (0.090) |  |  |  |
| Poverty Q3 |  | 1 (0.300) |  |  |  |
| Poverty Q2 |  | 1 (0.221) |  |  |  |

### Odds ratios and significance (Fig A of S1 Appendix)

|  | **New Zealand** | **Slovenia** | **Sweden** |
| --- | --- | --- | --- |
| Previous PTB | 6.3*** | 4.5*** | 7.1*** |
| Diabetes (chronic) |  | 3.3*** | 4.6*** |
| Nulliparous | 1.9*** | 1.6*** | 2.4*** |
| Diabetes (gestational) | 2*** | 1.7*** | 0.8** |
| Previous CS | 1.5*** | 1.9*** | 1.4*** |
| Preeclampsia | 1.8*** | 1.7*** | 1.4*** |
| ART |  | 1.8*** | 1.3*** |
| Hypertension (chronic) |  | 1.7*** | 0.6* |
| Maternal Age |  |  |  |
| Age > 40 | 1.3** | 1.7*** | 1.5*** |
| Age 35-40 | 1.2*** | 1.4*** | 1.2*** |
| Age < 20 | 1.1 (0.162) | 1.1 (0.150) | 0.9** |
| BMI |  |  |  |
| BMI (underweight) |  | 1.4*** | 1.3*** |
| BMI (overweight) |  | 1 (0.679) | 1.1*** |
| BMI (obese – class I) |  | 1.1 (0.094) | 1.2*** |
| BMI (obese – class II & III) |  | 1 (0.715) | 1.4*** |
| Smoking | 1.5*** | 1.3*** | 1.3*** |
| Male baby | 1.3*** | 1.2*** | 1.2*** |
| Education |  |  |  |
| High school or less |  | 1.2*** | 1.2*** |
| High school grad (or equ.) |  | 1 (0.139) | 1.1*** |
| Some college |  | 0.9 (0.309) | 1 (0.296) |
| Ethnicity |  |  |  |
| Ethnicity (Pacific P) | 0.8*** |  |  |
| Ethnicity (MELAA) | 1.1 (0.575) |  |  |
| Ethnicity (Asian) | 1 (0.689) |  |  |
| Ethnicity (Maori) | 1 (0.889) |  |  |
| Unemployed |  |  | 1 (0.050) |
| Hypertension (gestational) |  | 1.2 (0.052) | 0.3*** |
| No prenatal care before 20wk | 1.1 (0.057) | 1.1 (0.299) |  |
| Migration foreign born |  |  | 1 (0.676) |
| Single |  | 1 (0.364) | 1 (0.899) |
| Poverty |  |  |  |
| Poverty Q5 | 1.1 (0.073) |  |  |
| Poverty Q4 | 1 (0.251) |  |  |
| Poverty Q3 | 1.1 (0.194) |  |  |
| Poverty Q2 | 1 (0.842) |  |  |

### Odds ratios and significance (Fig B of S1 Appendix)

|  | **New Zealand** | **Slovenia** | **Sweden** |
| --- | --- | --- | --- |
| Previous PTB | 5.3*** | 4.6*** | 4.8*** |
| Preeclampsia | 4.4*** | 3.3*** | 5.2*** |
| Hypertension (chronic) |  | 2.8*** | 2.3*** |
| Hypertension (gestational) |  | 2.8*** | 0.8* |
| Diabetes (chronic) |  | 1.1 (0.592) | 2.4*** |
| Diabetes (gestational) | 1.6*** | 0.8 (0.123) | 0.9* |
| Smoking | 1.7*** | 1.1 (0.166) | 1.4*** |
| Nulliparous | 0.6*** | 1.6*** | 1.4*** |
| Maternal Age |  |  |  |
| Age > 40 | 1 (0.957) | 1.5** | 1.1 (0.126) |
| Age 35-40 | 1 (0.944) | 1.3*** | 1 (0.092) |
| Age < 20 | 1.1* | 0.9 (0.678) | 1.1 (0.108) |
| ART |  | 1.5*** | 1.2*** |
| Education |  |  |  |
| High school or less |  | 1.1 (0.064) | 1.3*** |
| High school grad (or equ.) |  | 0.9 (0.052) | 1.2*** |
| Some college |  | 0.9 (0.514) | 1.1*** |
| BMI |  |  |  |
| BMI (underweight) |  | 1.4** | 1.4*** |
| BMI (overweight) |  | 0.8*** | 0.9*** |
| BMI (obese – class I) |  | 0.7*** | 0.9*** |
| BMI (obese – class II & III) |  | 0.6*** | 1 (0.865) |
| No prenatal care before 20 wk | 1.1* | 1.1 (0.432) |  |
| Migration foreign born |  |  | 1.1* |
| Poverty |  |  |  |
| Poverty Q5 | 1.1* |  |  |
| Poverty Q4 | 1.1 (0.093) |  |  |
| Poverty Q3 | 1 (0.962) |  |  |
| Poverty Q2 | 1.1 (0.100) |  |  |
| Ethnicity |  |  |  |
| Ethnicity (Asian) | 0.9** |  |  |
| Ethnicity (Maori) | 1.1 (0.055) |  |  |
| Ethnicity (MELAA) | 1 (0.969) |  |  |
| Ethnicity (Pacific P) | 1 (0.600) |  |  |
| Previous CS | 0.5*** | 0.9 (0.431) | 0.8*** |
| Single |  | 0.9* | 1 (0.980) |
| Male baby | 1.1 (0.055) | 1 (0.455) | 1 (0.910) |
| Unemployed |  |  | 1 (0.063) |

### Odds ratios and significance (Fig C of S1 Appendix)

|  | **Czech Republic** | **New Zealand** | **Slovenia** | **Sweden** | **California** |
| --- | --- | --- | --- | --- | --- |
| Previous PTB | 5.3*** | 5.8*** | 4.5*** | 5.7*** | 7.8*** |
| Preeclampsia | 7*** | 3.7*** | 3.4*** | 9.2*** | 5*** |
| Hypertension (chronic) |  |  | 2.6*** | 3*** | 4.1*** |
| Ethnicity |  |  |  |  |  |
| Ethnicity (Non-Hispanic Black) |  |  |  |  | 2.6*** |
| Ethnicity (Other) |  |  |  |  | 2*** |
| Ethnicity (Hispanic) |  |  |  |  | 1.4*** |
| Ethnicity (Asian) |  | 1.1 (0.361) |  |  | 1.4*** |
| Ethnicity (MELAA) |  | 1.4 (0.054) |  |  |  |
| Ethnicity (Maori) |  | 1.2*** |  |  |  |
| Ethnicity (Pacific P) |  | 1.1 (0.105) |  |  |  |
| Maternal age |  |  |  |  |  |
| Age > 40 | 2.5*** | 1.3 (0.063) | 2.1*** | 1.6*** | 1.8*** |
| Age 35-40 | 1.7*** | 1.1 (0.069) | 1.6*** | 1.4*** | 1.5*** |
| Age < 20 | 1 (0.992) | 1.1 (0.090) | 1.2 (0.274) | 0.9 (0.570) | 1 (0.266) |
| Nulliparous | 1.6*** | 1.6*** | 2*** | 2.4*** | 1.6*** |
| ART |  |  | 2.2*** | 1.8*** | 2.3*** |
| Diabetes (chronic) | 2.2*** |  | 1.6 (0.12771) | 1.8*** |  |
| Drug use (illicit) | 1.8*** |  |  |  |  |
| Education |  |  |  |  |  |
| Less than high school grad | 1.8*** |  | 1.5*** | 1.3*** | 1.7*** |
| High school grad but no college | 1.3*** |  | 0.9 (0.415) | 1.1* | 1.5*** |
| Some college | 1.1* |  | 0.8 (0.072) | 1.1* | 1.4*** |
| Smoking | 1.4*** | 1.7*** | 1.3*** | 1.6*** | 1.5*** |
| Hypertension (gestational) | 1 (0.622) |  | 1.6*** | 0.5** |  |
| Previous CS | 1.3*** | 1.1 (0.094) | 1.4*** | 1.4*** | 1.3*** |
| BMI |  |  |  |  |  |
| BMI (underweight) |  |  | 1.4** | 1.1 (0.09741) | 1.2*** |
| BMI (overweight) |  |  | 1 (0.904) | 1.1*** | 1.1*** |
| BMI (obese – class I) |  |  | 1.1 (0.273) | 1.3*** | 1.3*** |
| BMI (obese – class II & III) |  |  | 0.8 (0.183) | 1.7*** | 1.5*** |
| No prenatal care before 20 wk | 1.3*** | 1.5*** | 0.7 (0.094) |  | 0.9 (0.054) |
| Poverty |  |  |  |  |  |
| Poverty Q5 |  | 1.3*** |  |  |  |
| Poverty Q4 |  | 1.2* |  |  |  |
| Poverty Q3 |  | 1.1 (0.186) |  |  |  |
| Poverty Q2 |  | 1.2 (0.063) |  |  |  |
| Migration foreign born |  |  |  | 1.2*** | 0.8*** |
| Male baby | 1.2*** | 1.2*** | 1.1* | 1.2*** | 1.2*** |
| Single | 1.2*** |  | 0.7*** | 1.1 (0.164) |  |
| Unemployed |  |  |  | 0.9*** |  |
| Diabetes (gestational) | 0.9* | 1 (0.910) | 0.6** | 0.5*** | 1 (0.234) |

### 95% confidence intervals (Fig 3)

|  | **Czech Republic** | **New Zealand** | **Slovenia** | **Sweden** | **California** |
| --- | --- | --- | --- | --- | --- |
| Previous PTB | 5.048-5.348 | 5.322-6.047 | 4.187-4.950 | 5.810-6.222 | 4.737-5.243 |
| Preeclampsia | 4.578-5.029 | 3.259-3.644 | 2.377-3.192 | 5.436-5.916 | 4.080-4.323 |
| Diabetes (chronic) | 3.012-3.734 |  | 1.423-2.499 | 3.371-3.942 |  |
| Hypertension (chronic) |  |  | 1.792-2.448 | 1.372-2.029 | 2.850-3.229 |
| Maternal Age |  |  |  |  |  |
| Age > 40 | 1.645-1.881 | 1.140-1.418 | 1.357-1.868 | 1.325-1.499 | 1.480-1.581 |
| Age 35-40 | 1.342-1.417 | 1.112-1.229 | 1.270-1.455 | 1.157-1.223 | 1.240-1.290 |
| Age < 20 | 1.028-1.107 | 1.014-1.159 | .951-1.338 | 0.835-0.971 | 0.962-1.013 |
| Nulliparous | 1.514-1.567 | 1.359-1.472 | 1.512-1.663 | 2.064-2.155 | 1.138-1.175 |
| ART |  |  | 1.529-1.896 | 1.266-1.369 | 1.526-1.881 |
| Drug use (illicit) | 1.468-1.878 |  |  |  |  |
| Ethnicity |  |  |  |  |  |
| Ethnicity (other) |  |  |  |  | 1.596-1.706 |
| Ethnicity (Non-Hispanic Black) |  |  |  |  | 1.548-1.638 |
| Ethnicity (Asian) |  | 0.919-1.037 |  |  | 1.231-1.304 |
| Ethnicity (Hispanic) |  |  |  |  | 1.179-1.226 |
| Ethnicity (Pacific P) |  | 0.820-0.945 |  |  |  |
| Ethnicity (MELAA) |  | 0.951-1.241 |  |  |  |
| Ethnicity (Maori) |  | 0.947-1.040 |  |  |  |
| Smoking | 1.268-1.340 | 1.513-1.664 | 1.216-1.383 | 1.272-1.350 | 1.312-1.417 |
| Diabetes (gestational) | 1.264-1.374 | 1.763-2.052 | 1.169-1.446 | 0.777-0.949 | 1.249-1.331 |
| Hypertension (gestational) | 1.212-1.341 |  | 1.415-1.811 | 0.502-0.714 |  |
| BMI |  |  |  |  |  |
| BMI (underweight) |  |  | 1.323-1.563 | 1.235-1.376 | 1.218-1.302 |
| BMI (overweight) |  |  | .864-.971 | 1.017-1.063 | 0.969-1.002 |
| BMI (obese – class I) |  |  | .839-1.006 | 1.108-1.184 | 1.015-1.060 |
| BMI (obese – class II & III) |  |  | .649-.873 | 1.254-1.375 | 1.057-1.112 |
| Education |  |  |  |  |  |
| Less than high school grad | 1.406-1.493 |  | 1.127-1.286 | 1.203-1.267 | 1.315-1.381 |
| High school grad but no college | 1.032-1.084 |  | .960-1.063 | 1.080-1.134 | 1.226-1.281 |
| Some college | 1.000-1.046 |  | .861-1.030 | 1.024-1.089 | 1.193-1.245 |
| Previous CS | 1.179-1.252 | 1.118-1.258 | 1.104-1.335 | 1.274-1.361 | 1.273-1.323 |
| Male baby | 1.153-1.188 | 1.162-1.246 | 1.116-1.214 | 1.126-1.169 | 1.159-1.191 |
| No prenatal care before 20 wk | 1.651-1.777 | 1.040-1.168 | 0.971-1.263 |  | 1.090-1.155 |
| Single | 1.145-1.184 |  | 0.886-0.995 | 0.973-1.056 |  |
| Unemployed |  |  |  | 0.939-0.988 |  |
| Migration foreign born |  |  |  | 0.992-1.044 | 0.835-0.864 |
| Poverty |  |  |  |  |  |
| Poverty Q5 |  | 1.012-1.143 |  |  |  |
| Poverty Q4 |  | 0.992-1.116 |  |  |  |
| Poverty Q3 |  | 0.972-1.096 |  |  |  |
| Poverty Q2 |  | .977-1.106 |  |  |  |

# Additional references

1. "BMI Classification". Global Database on Body Mass Index. . World Health Organization. 2006.

2. Bloch M, Althabe F, Onyamboko M, Kaseba-Sata C, Castilla EE, Freire S, et al. Tobacco use and secondhand smoke exposure during pregnancy: an investigative survey of women in 9 developing nations. Am J Public Health. 2008;98(10):1833-40.

3. Chang HH, Larson J, Blencowe H, Spong CY, Howson CP, Cairns-Smith S, et al. Preventing preterm births: analysis of trends and potential reductions with interventions in 39 countries with very high human development index. Lancet. 2013;381(9862):223-34.

4. Smedberg J, Lupattelli A, Mardby AC, Nordeng H. Characteristics of women who continue smoking during pregnancy: a cross-sectional study of pregnant women and new mothers in 15 European countries. BMC Pregnancy Childbirth. 2014;14:213.

5. Schneider S, Maul H, Freerksen N, Potschke-Langer M. Who smokes during pregnancy? An analysis of the German Perinatal Quality Survey 2005. Public Health. 2008;122(11):1210-6.

6. Mensink GB, Schienkiewitz A, Haftenberger M, Lampert T, Ziese T, Scheidt-Nave C. [Overweight and obesity in Germany: results of the German Health Interview and Examination Survey for Adults (DEGS1)]. Bundesgesundheitsblatt Gesundheitsforschung Gesundheitsschutz. 2013;56(5-6):786-94.

7. al-Mannai A, Dickerson JW, Morgan JB, Khalfan H. Obesity in Bahraini adults. J R Soc Health. 1996;116(1):30-2, 7-40.

8. al. MJMe. National Registry of Hypertension. Awareness, Treatment and Control of Hypertension. The RENATA Study. REVISTA ARGENTINA DE CARDIOLOGÍA. 2012;80(2).

9. Neuhauser H, Thamm M, Ellert U. [Blood pressure in Germany 2008-2011: results of the German Health Interview and Examination Survey for Adults (DEGS1)]. Bundesgesundheitsblatt Gesundheitsforschung Gesundheitsschutz. 2013;56(5-6):795-801.

10. Miura K, Nagai M, Ohkubo T. Epidemiology of Hypertension in Japan. Circulation Journal. 2013;77(9):2226-31.

11. Abdulbari Bener ea. The prevalence of hypertension and its associated risk factors in a newly developed country. Saudi Med Journal. 2004;25(7):918-22.

12. Cifkova R, Skodova Z, Lanska V, Adamkova V, Novozamska E, Jozifova M, et al. Prevalence, awareness, treatment, and control of hypertension in the Czech Republic. Results of two nationwide cross-sectional surveys in 1997/1998 and 2000/2001, Czech Post-MONICA Study. J Hum Hypertens. 2004;18(8):571-9.

13. Ann-Britt E. Wiréhn HMK, John M. Carstensen. Estimating disease prevalence using a population-based administrative healthcare database. Scand J Public Health. 2007;35(4):424-31.

14. Roberts CL, Ford JB, Algert CS, Antonsen S, Chalmers J, Cnattingius S, et al. Population-based trends in pregnancy hypertension and pre-eclampsia: an international comparative study. BMJ Open. 2011;1(1):e000101.

15. Bouvier-Colle MH, Salanave B, Ancel PY, Varnoux N, Fernandez H, Papiernik E, et al. Obstetric patients treated in intensive care units and maternal mortality. Regional Teams for the Survey. Eur J Obstet Gynecol Reprod Biol. 1996;65(1):121-5.

16. Schneider S, Freerksen N, Maul H, Roehrig S, Fischer B, Hoeft B. Risk groups and maternal-neonatal complications of preeclampsia--current results from the national German Perinatal Quality Registry. J Perinat Med. 2011;39(3):257-65.

17. Bener A, Saleh NM, Al-Hamaq A. Prevalence of gestational diabetes and associated maternal and neonatal complications in a fast-developing community: global comparisons. Int J Womens Health. 2011;3:367-73.

18. KH Tan KK, GS Yeo. Epidemiology of pre-eclampsia and eclampsia at the KK Women's and Children's Hospital, Singapore. Singapore Med J. 2006;47(1).

19. Ananth CV, Keyes KM, Wapner RJ. Pre-eclampsia rates in the United States, 1980-2010: age-period-cohort analysis. BMJ. 2013;347:f6564.

20. Ishihara O, Adamson GD, Dyer S, de Mouzon J, Nygren KG, Sullivan EA, et al. International committee for monitoring assisted reproductive technologies: world report on assisted reproductive technologies, 2007. Fertil Steril. 2015;103(2):402-13 e11.
